# Does propranolol have a role in cancer treatment? A systematic review of the epidemiological and clinical trial literature on beta-blockers

**DOI:** 10.1007/s00432-025-06262-2

**Published:** 2025-07-12

**Authors:** Jessica O’Logbon, Ludovica Tarantola, Norman R. Williams, Shreeya Mehta, Aamir Ahmed, Elizabeth A. Davies

**Affiliations:** 1 Guy’s and St. Thomas’ NHS Trust, St Thomas’ Hospital, Westminster Bridge Rd, London, SE1 7EH UK; 2 GKT School of Medical Education, Guy’s Campus, Great Maze Pond London, London, SE1 1UL UK; 3 Prostate Cancer Research Centre at the Centre for Stem Cell and Regenerative Medicine, 28 Floor Guy’s Hospital, King’s College London, Great Maze Pond, London, SE1 9RT UK; 4 Division of Surgery & Interventional Science, University College London, 43-45 Foley, London, W1W 7TY UK; 5 Oncolodyne Ltd., Rockefeller Building, University College London, 21 University Street, London, WC1E 6DE UK; 6 Cell and Developmental Biology, Rockefeller Building, University College London, 21 University Street, London, WC1E 6DE UK; 7 Comprehensive Cancer Centre, 3rd Floor Bermondsey Wing, Guy’s Hospital, King’s College London, London, SE1 7LB UK

**Keywords:** Beta-blockers, Propranolol, Cancer treatment, Systematic reviews, Repurposing

## Abstract

**Purpose:**

Beta-blockers, originally developed for cardiovascular conditions, have been explored for their potential role in cancer treatment. Propranolol, a non-selective beta-blocker, has shown promise in inhibiting stress-induced signalling pathways associated with cancer progression. This systematic review aims to assess the evidence for the repurposing of propranolol as a treatment for various cancers, particularly breast cancer to answer the research question: *Does propranolol improve cancer outcomes, including survival and recurrence?*

**Methods:**

We conducted a systematic search of MEDLINE, EMBASE, Global Health, Web of Science, and the Cochrane Library, including studies up to July 2024. Randomised Controlled Trials (RCTs), systematic reviews, and meta-analyses were included if they assessed the effects of propranolol on cancer outcomes such as mortality, survival, recurrence, or biomarkers of tumour regression. A narrative synthesis was performed to summarise the findings.

**Results:**

Thirty-one studies were included, consisting of 7 RCTs, 4 systematic reviews and 20 meta-analyses. The evidence suggests that propranolol may improve cancer outcomes, especially when administered perioperatively, by reducing recurrence risk. However, the results remain inconclusive regarding its use in combination with chemotherapy or radiotherapy, as studies showed mixed results. The timing of propranolol administration, alongside its combination with other cancer therapies, appears to be a key factor in its effectiveness.

**Conclusion:**

Propranolol has potential as an adjunctive therapy in cancer treatment, particularly in reducing recurrence risk during the perioperative period. However, further clinical trials are needed to better define its role in cancer therapy, particularly regarding optimal treatment regimens and patient populations.

**Supplementary Information:**

The online version contains supplementary material available at 10.1007/s00432-025-06262-2.

## Introduction

The lengthy process of developing new cancer therapies may be expedited through drug repurposing or repositioning, which involves identifying new therapeutic applications for existing medications (Issa et al. 2013; Xia et al. 2024). One example is rapamycin (also known as sirolimus), approved in 1999, which was initially developed as an immunosuppressant to prevent rejection in kidney transplant patients (Tanaka et al. 1987). The biological target of rapamycin is the eponymous mammalian target of rapamycin complex 1 (mTORC1), a key pathway that regulates cell growth, proliferation, survival, and metabolism. It was later discovered that dysregulation of this mTOR pathway is common in many cancers, contributing to abnormal cell growth, metabolism, and resistance to cell death (Fasolo and Sessa 2012). This led rapamycin and related mTOR inhibitors (like everolimus and temsirolimus) to be repurposed for cancer therapy, particularly where mTOR is highly active, such as renal cell carcinoma and certain types of breast and neuroendocrine tumours (Dancey 2010). Another example is indomethacin, a non-steroidal anti-inflammatory drug (NSAID), widely used to modulate inflammatory responses and has been found to suppress cancer cell migration by inhibiting calcium influx and focal complex formation (Guo et al. 2013).

In recent years, beta-blockers, traditionally used for cardiovascular conditions like hypertension and arrhythmias, have shown promise as potential anti-cancer agents and been proposed to improve survival for several types of cancer (Cavalu et al. 2024; Peixoto et al. 2020). These drugs block beta-adrenergic receptors, reducing the amount of endogenous beta agonists (such as catecholamines) in the body, which are associated with mechanisms that trigger tumorigenesis, angiogenesis, and tumour metastasis (Carnet Le Provost et al. 2023). These mechanisms include the activation of inflammation and of genes associated with metastasis, cell proliferation pathways and upregulation of pro-angiogenic factor and vascular epithelia growth factor (VEGF) (Carnet Le Provost et al. 2023).

Considering that mediation via beta-2 receptors seems to be partly responsible for those mechanisms, a non-selective beta-1 and beta-2 receptor antagonist like propranolol should be a more promising potential anti-cancer agent than selective beta-1 receptor antagonists (Cole and Sood 2011). Propranolol has therefore attracted particular interest due to its ability to inhibit stress-induced signalling pathways that are active in various cancers, including breast, melanoma, and pancreatic cancers (Pantziarka et al. 2018). In vitro studies show that exposure of prostate and breast cancer cells to propranolol induces membrane currents and intracellular calcium (Ca^2+^_i_) release, a key second messenger in various cellular processes, such as proliferation, differentiation, apoptosis, and gene transcription (Berridge et al. 2000; Petrou et al. 2017; Reyes-Corral et al. 2019; Weiss et al. 2013). Ca^2+^_i_ is also critical for Wnt signalling which in turn is associated with cancers of the colon, breast, prostate, and skin, as well as developmental disorders and skeletal diseases (Howe and Brown 2004; Li et al. 2015; Logan and Nusse 2004; Schatoff et al. 2017; Wang et al. 2010).

Most notably, propranolol is effective in the treatment of haemangioma, a rare benign tumour diagnosed in infants and children (Holmes et al. 2011). A surprisingly consistent rate of regression and reduced size, arrest of the proliferation phase and decrease of VEGF has been reported in these tumours (Holmes et al. 2011; Malik et al. 2013; Vercellino et al. 2013). Trials have shown that propranolol produced a better and faster response compared with control treatment (Sondhi and Patnaik 2013; Léauté-Labrèze et al. 2015) but it is not yet clear how propranolol causes these beneficial effects.

Regarding cancerous tumours, evidence for anti-metastatic effects of propranolol comes from a range of investigators pursuing several lines of both preclinical and clinical research. A team at Texas Tech University Health Sciences Centre retrospectively assessed the impact of selective and non-selective beta-blockers on tumour proliferation (Ki67) (Montoya et al. 2017, 2019). Results showed that non-selective beta blockade reduced tumour proliferation by 66% in early-stage breast cancer and cell line data showed that propranolol dose dependently reduced tumour cell viability (Montoya et al. 2017). Retrospective studies have shown that beta-blocker usage is associated with improved recurrence free survival in women with triple-negative breast cancer (Botteri et al. 2013; Melhem-Bertrandt et al. 2011) and reduced risk of metastasis (Botteri et al. 2013).However, the evidence that propranolol is effective in breast cancer is still limited and few systematic reviews or meta-analyses have analysed data from randomised controlled trials. A recent meta-analysis concluded that the use of propranolol did not cause any significant difference in cancer specific death rate, overall death rate or relapse-free survival rate between compared to those who did not (Kim et al. 2017). With emerging evidence of its role in membrane potential regulating activity (Reyes-Corral et al. 2019) and the role membrane potential plays in key cancer signalling pathways, such as the Wnt pathway (Ashmore et al. 2019), we believe a re-analysis of propranolol in cancer treatment is necessary.

Unlike previous reviews on this subject (Caparica et al. 2021; Scott et al. 2024) that have analysed epidemiological and clinical evidence for beta-blockers as a class or limited to breast cancer, our systematic review is the first to isolate and evaluate propranolol specifically, offering a detailed synthesis of both propranolol trials and mechanistic biomarkers in breast cancer and comparing the outcomes with other cancers. Our primary aim was to determine whether there is substantial evidence for the repurposing of beta-blockers, specifically propranolol, for breast cancer treatment as well as other cancers. The objectives were to (i) interrogate published systematic reviews and meta-analyses to ensure the inclusion of as many studies as possible and (ii) review the most up-to-date randomised controlled trials to determine whether there is more conclusive evidence that these drugs influence cancer outcomes including incidence, recurrence and survival together with biomarkers of tumour regression or death.

## Methods

### Design

This systematic review was conducted following Cochrane methodology and Preferred Reporting Items for Systematic Reviews and Meta-analyses (PRISMA) guidelines (Page et al. 2021). The review was registered with PROSPERO in July 2024 (CRD42024568054).

### Search strategy

MEDLINE, EMBASE, Global Health, Web of Science and the Cochrane Library were searched from database inception to 1 July 2024 to identify peer-reviewed studies in English. The search strategy was developed using the patient, intervention, comparator and outcome (PICO) framework. The primary terms for the search strategy were related to the following keywords: ‘‘beta-blocker”, “propranolol” and “cancer”. Boolean operators were used to connect specific search keywords for each database and other free text terms. Search terms were based on a preliminary search of the relevant literature and reviewed and approved by the authors. The specific rules and vocabulary of each database were used, and the search strategy was designed by one author (JO) and discussed with two other authors (NRW and EAD).

No limits were placed on the dates that papers were published, in order to capture any studies which previous reviews might have missed. We combined the following search terms and their associated wildcard variants using Boolean operators:

- “Beta blockers”, “propranolol”
- “Cancer”, “tumour”, “oncology”, or “malignancy”– defined as the presence of cancerous cells that have the ability to spread to other sites in the body (metastasise) or to invade nearby (locally) and destroy tissues. Benign tumours were excluded.
- “Clinical trials”, “meta-analyses”, “randomised controlled trial”, “review”, “systematic review”

The search terms within groups were combined with ‘OR’ whereas each domain was combined with ‘AND’. The full search strategy can be found in Supplemental Information (Online Resource 1).

### Eligibility criteria

A randomised controlled trial was included if it fulfilled the following, based on the PICO criteria:

1. *Population* Patients of all ages diagnosed with cancer. The diagnosis should have been established using standard criteria, including imaging and lab tests if indicated, and this should be described in the paper. Patients with benign tumours, such as haemangiomas, were not eligible.
2. *Intervention* The use of propranolol, including as an adjunct to current cancer treatments like chemo/radiotherapy or peri-operatively, in patients before or after their cancer diagnosis.

Studies were ineligible if:

- Beta-blockers were used in the intervention arm with no mention of propranolol specifically or results were not reported separately for it. Although, we may comment on the results of such papers in the discussion.
- *Comparison* Placebo, treatment as usual (e.g. cancer treatment), alternative medications including comparison to other beta-blockers or anti-hypertensives.
- *Outcomes*
  - The effect of beta-blockers on mortality, survival, recurrence or incidence/risk in cancer patients including genes or biomarkers of tumour regression or apoptosis.
  - In the case of meta-analyses or systematic reviews, reported hazard ratio (HR), relative risk (RR) and 95% confidence intervals (CI).

Papers were excluded if they were not available in English, did not have human participants and were of other publication types.

## Study selection and data extraction

Duplicate references were first removed in Mendeley reference manager, and titles and abstracts of articles were independently reviewed for eligibility by JO and NRW using the software Rayyan (). Conflicts in decisions made were reasoned and discussed. A Cohen’s Kappa of 0.6915 was calculated, suggesting substantial agreement between screeners. Data extraction was carried out by two authors (JO and SM) using the Cochrane Data Extraction and Assessment form as a guide. The following data were extracted: population characteristics including demographics, eligibility criteria and trial arms, cancer outcomes measured (primary and secondary endpoints as reported in studies), the methods of statistical analyses used, results and length of follow-up (as ranges, medians or means as reported in studies).

The Cochrane risk of bias tool (Higgins et al. 2011) was used to assess risk of bias in the RCTs.

## Results

**Fig. 1:**
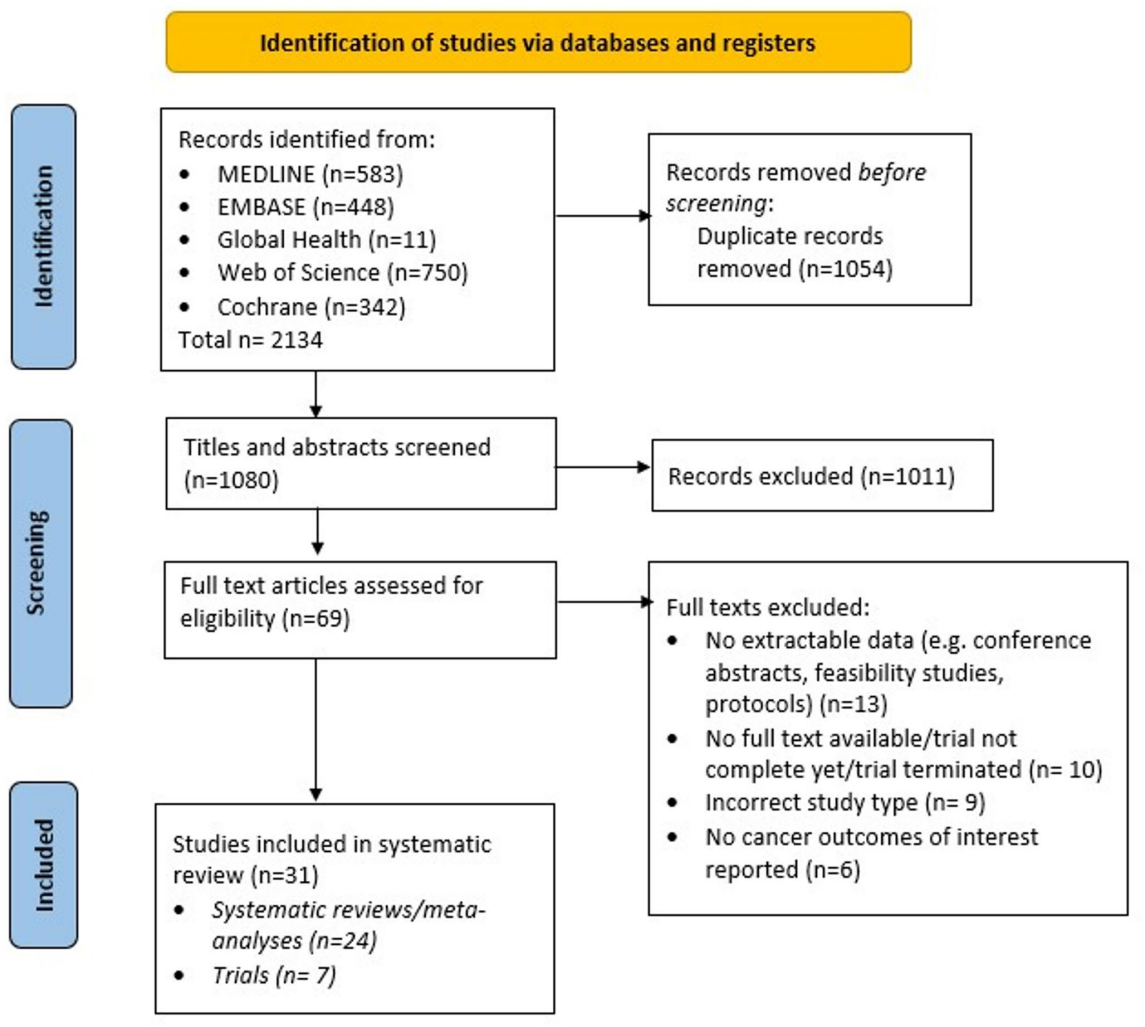
PRISMA flow chart of included studies

The PRISMA flow chart for the review search is summarised in Fig. 1.

A total of 2134 studies were initially identified with 1054 being excluded as duplicates and a further 1011 excluded after screening titles and abstracts as not meeting the inclusion criteria.

Sixty-nine studies were eligible for full-text screening and after retrieval and evaluation, 38 were excluded, according to criteria described in Fig. 1. The remaining 31 articles were used for this systematic review, of which 24 were systematic reviews or meta-analyses and 7 were randomised controlled/clinical trials.

### Breast cancer

Table 1 shows the characteristics of studies and reviews that were included which evaluated beta-blockers and breast cancer outcomes (n = 17). Five randomised controlled trials studied the use of propranolol in breast cancer (Haldar et al. 2018; Hiller et al. 2020; Shaashua et al. 2017; Spera et al. 2017; Zhou et al. 2016) of which four (Haldar et al. 2018; Hiller et al. 2020; Shaashua et al. 2017; Zhou et al. 2016) investigated the use of propranolol as an adjunct to surgical resection of a breast tumour.

**Table 1:** Characteristics and findings of studies and reviews that investigated the effects of beta blockers on breast cancer outcomes (n = 17)

Title	Author, year	Study type	Population characteristics	Cancer outcomes	Statistical analysis	Results/key findings	Length of follow-up
Beta-blockers in early-stage breast cancer: a systematic review and meta-analysis	Caparica et al. (2021)	Systematic review and meta-analysis	13 studies, n = 141,771	Primary endpoint = recurrence-free survival (RFS), defined as the occurrence of breast cancer recurrence or death. Secondary objectives were pathologic complete response (pCR), breast cancer recurrence, breast cancer-specific mortality and overall survival (OS)	Primary endpoints: hazard ratios (HRs) were extracted from each study to compare patients receiving or not receiving beta-blockers Secondary objectives pCR and breast cancer recurrence: odds ratios (ORs) were extracted from each study (when available), or calculated for the number of events occurring in patients who received beta-blocker versus those who did not. For each HR or OR estimate, 95% confidence intervals (CIs) were computed. Pooled HRs and ORs were calculated using the random-effects model. The Higgins’ I^2^ index was computed to obtain a quantitative measure of the degree of inconsistency in the results of the studies	Beta-blocker use was associated with a longer RFS in patients with early-stage breast cancer, with a more pronounced effect observed in triple-negative disease. Their use was also associated with a significant RFS improvement in the overall population (N = 21,570; HR 0.73; 95% CI, 0.56–0.96; *P* = 0.025) and in patients with triple-negative disease (N = 1212; HR 0.53; 95% CI, 0.35–0.81; *P* = 0.003). No significant differences were observed for pCR (N = 1554; OR 0.77; 95% CI 0.44–1.36; P = 0.371), breast cancer recurrence (N = 37 957; OR 0.66; 95% CI, 0.42–1.03; *P* = 0.065), breast cancer-specific mortality (N = 64,830; HR 0.77; 95% CI 0.56–1.08; *P* = 0.130) or OS (N = 103,065; HR 1.03; 95% CI, 0.87–1.23; P = 0.692) according to beta-blocker use	2 to 10.3 years
Propranolol and survival from breast cancer: a pooled analysis of European breast cancer cohorts	Cardwell et al. (2016)	Meta-analysis of cohort studies	8 European cohort studies pooled to include 55,252 and 133,251 breast cancer patients in the analysis of breast cancer-specific and all-cause mortality respectively. All cohorts identified incident invasive diagnoses using cancer registry data 1998 to 2012	In 7 of the cohorts, mortality was ascertained from national death records; social security records were used in one cohort. Breast cancer-specific mortality was defined as breast cancer being the underlying cause of death and was available in five cohorts. All-cause mortality was available in all cohorts	Time-dependent Cox regression models were used to calculate hazard ratios (HRs) and 95% confidence intervals (CIs) for breast cancer-specific death in propranolol users compared with propranolol non-users	No difference in risk was found between use after the diagnosis and subsequent breast cancer-specific (HR 0.94 (95% CI 0.77, 1.16) or all-cause mortality (HR 1.09 CI 0.93–1.28) No difference in risk was found between propranolol and non-selective* β*-blockers use before breast cancer diagnosis and breast cancer-specific mortality (HR 1.03,95%CI 0.86–1.22) or all-cause mortality (HR 1.02, 95% CI 0.94–1.10)	Maximum follow-up in each cohort after diagnosis ranged from 5 to 13 years
β-Blockers Reduce Breast Cancer Recurrence and Breast Cancer Death: A Meta-Analysis	Childers et al. (2015)	Systematic review and meta-analysis	7 studies, n = 28,784	End points: breast cancer recurrence, death from breast cancer, and all-cause mortality in patients receiving beta-blockers	Woolf test was used to test for heterogeneity and suggested statistically significant heterogeneity between studies (P =.022).25 To deal with heterogeneity between studies the authors used random effectsmodels to estimate pooled HRs. The method of Parmar et al. was used to estimate standard errors of HRs for individual studies	There was no statistically significant risk reduction (HR, 0.67; 95% confidence interval [CI], 0.39–1.13). Breast cancer deaths were recorded in 4 studies, which suggested a significant reduction in risk (HR, 0.50; 95% CI 0.32–0.80). Among the 4 studies that reported all-cause mortality, there was no significant effect of beta-blockers on risk (HR, 1.02; 95% CI 0.75–1.37). This systematic review and meta-analysis suggested that beta-blockers significantly reduced risk of breast cancer death among women with breast cancer	N/A
Perioperative inhibition of beta-adrenergic and COX2 signaling in a clinical trial in breast cancer patients improves tumor Ki-67 expression, serum cytokine levels, and PBMCs transcriptome	Haldar et al. (2018)	Clinical trial	38 patients newly diagnosed with breast cancer without known metastatic disease were recruited from three medical centres in Israel and randomly allocated to: control n = 19, propranolol + COX-2 inhibitor (etodolac n = 19) Age 33–70, mean = 55.3, SD = 8.71)	Transcription control pathways and whole genome mRNAprofiles (ii) Serum levelsof several cytokines and soluble factors (iii) Markers of proliferation and cancer progression in the tumour, using immunohistochemistry staining and mRNA profiling	Repeated measures analysis of variance (ANOVA) was used to assess group differences (drug treatment), time (T1–T4), and interaction, and Fisher’s PLSD post-hoc comparisons were performed to analyse group differences at specific time-points	Serum levels of pro-inflammatory IL-6, CRP, and IFNγ, and anti-inflammatory, cortisol and IL-10, increased Drug treatment reduced serum levels of these pro-inflammatory cytokines, as well as activity of multiple inflammation-related transcription factors, but not serum levels of cortisol, IL-10, IL-18, IL-8, VEGF and TNFα In the excised tumour, treatment reduced the expression of Ki-67 and positively affected its transcription factors SP1 and AhR Exploratory analyses of transcriptome modulation in PBMCs revealed treatment-induced improvement in several transcription factors	5 days post-operatively
Perioperative events influence cancer recurrence risk after surgery	Hiller et al. (2020)	Randomised controlled trial (Phase II)	N = 60, English-speaking female patients aged 18 to 80 years (Eastern Cooperative Oncology Group < 2) with a diagnosis of surgically resectable primary breast cancer were eligible Intervention arm: 53.2 years (SD = 9.5), Control arm: 56.6 years (SD = 9.3) Oral propranolol (n = 30; 80–160 mg daily) vs placebo (n = 30) starting 7 days prior to the date of surgery and weaned off 3 days after surgery	The effect of propranolol on pro-metastatic and pro-inflammatory gene expression within the primary tumour	Per protocol analysis Quantitative change in tumour tissue pro-metastatic gene expression from baseline biopsy to resected primary tumour (per protocol and ITT)	Propranolol downregulated primary tumour expression of mesenchymal genes (*P* = 0.002) without affecting epithelial gene expression (*P* = 0.21). One week of beta-blockade with propranolol reduced intra-tumoral mesenchymal polarization and promoted immune cell infiltration in early-stage surgically resectable breast cancer	After surgery, patients were weaned from study medication over 3 days
β-Blocker use is not associated with improved clinical outcomes in women with breast cancer: a meta-analysis	Li et al. (2020)	Meta-analysis	17 observational studies including 75,074 women. Those with beta-blocker use 1 year prior to the diagnosis or after the diagnosis of breast cancer was considered as beta-blocker users. Age ranged 49—76 years	Prognosis of breast cancer patients using beta blockers	Random-effect model was used to pool the results	Pooled results did not support a significant association between beta-blocker use and breast cancer recurrence (risk ratio [RR] = 0.85, 95% confidence interval [CI]: 0.68–1.07, *P* = 0.17), breast cancer related deaths (RR = 0.83, 95% CI 0.65–1.06, *P* = 0.14), or all-cause deaths (RR = 1.01, 95% CI 0.91–1.11, *P* = 0.91) in women with breast cancer Subgroup analyses showed that beta-blockers may be associated with a trend of reduced risk of all-cause deaths in prospective studies (two datasets, RR = 0.81, *p*= 0.05), but not in retrospective studies (eight datasets, RR = 1.06, *P* = 0.16; P for subgroup analyses = 0.02)	The mean follow-up duration varied from 2.1 to 10.5 years
β-blockers and breast cancer survival by molecular subtypes: a population-based cohort study and meta-analysis	Løfling et al. (2022)	Population-based cohort study and meta-analysis of observational studies	30,060 women aged ≥ 50 years with breast cancer diagnosed between 2004 and 2018 in Norway, of which 4461 (15%) used beta-blockers	Assess the association between use of beta-blockers at the time of diagnosis and breast cancer -specific survival	Cox regression models to estimate the association between beta-blocker use at diagnosis and breast cancer -specific survival, overall and by molecular subtype. Complete-case analysis including only women who had no missing value for any of the adjustment variables and a multiple imputation analysis	Overall, beta-blocker use was not associated with breast cancer -specific survival (hazard ratio [HR] = 1.07; 95% confidence interval [CI]: 0.97–1.19). There was an association only in triple-negative breast cancer) patients (HR = 0.66; 95% CI 0.47–0.91). This was confirmed in the meta-analysis: beta-blocker use was associated with progression/recurrence-free (HR = 0.58; 95% CI 0.38–0.89) and BC-specific survival (HR = 0.74; 95% CI 0.55–1.00) in TNBC patients only	Median follow-up of 5.1 years
The Influence of Pre-Existing Beta-Blockers Use on Survival Outcomes in HER2 Positive Advanced Breast Cancer: Pooled Analysis of Clinical Trial Data	Modi et al. (2020)	Meta-analysis	Data from clinical trials EMILIA, TH3RESA, MARIANNE, and CLEOPATRA was pooled, n = 2777	The association of pre-existing beta blocker use with overall survival (PFS assessed as a secondary outcome) whilst initiating anti-HER2 therapy for advanced breast cancer	Cox proportional hazard analysis was used to assess the association between pre-existing or concomitant beta blocker use with overall survival and progression free survival. Results were reported as hazard ratios (HR) with 95% confidence intervals (95%CI). Kaplan–Meier analysis was used for plotting and estimating these probabilities	Beta blocker use was associated with worse overall survival (adjusted HR = 1.27, 95% CI 1.04–1.55). No statistically significant association between their use and progression free survival was identified (adjusted HR = 1.10, 95% CI 0.92–1.30)	Median (Interquartile Range) follow-up was 50 [95% CI 49–51] months in CLEOPATRA, 35 [34–36] months in MARIANNE, 47 [46–49] months in EMILIA and 35 [34–36] months in TH3RESA
Beta-Blocker Drug Therapy Reduces Secondary Cancer Formation in Breast Cancer and Improves Cancer Specific Survival	Powe et al. (2010)	Systematic review	The combined study population included 55,252 and 133,251 breast cancer patients in the analysis of breast cancer-specific and all-cause mortality respectively	In seven of the cohorts, mortality was ascertained from national death records; social security records were used in one cohort. Breast cancer-specific mortality was defined as breast cancer being the underlying cause of death and was available in five cohorts. All-cause mortality was available in all cohorts	Cox regression models were used to calculate hazard ratios (HR) and 95% confidence intervals (CIs) for cancer- specific and all-cause mortality by propranolol and non-selective beta-blocker use. HRs were pooled across cohorts using meta-analysis techniques. Dose–response analyses by number of prescriptions were also performed	Overall, there was no association between propranolol use after the diagnosis of breast cancer and breast cancer-specific or all-cause mortality (fully adjusted HR = 0.94, 95% CI, 0.77, 1.16 and HR = 1.09, 95% CI, 0.93, 1.28, respectively). There was little evidence of a dose–response relationship. There was also no association between propranolol use before breast cancer diagnosis and breast cancer-specific or all-cause mortality (fully adjusted HR = 1.03, 95% CI, 0.86, 1.22 and HR = 1.02, 95% CI, 0.94, 1.10, respectively)	Mean follow-up 4–6 years. The maximum follow-up in each cohort after diagnosis of breast cancer ranged from 5 to 13 years
Use of beta-blockers, angiotensin-converting enzyme inhibitors and angiotensin receptor blockers and breast cancer survival: Systematic review and meta-analysis	Raimondi et al. (2016)	Systematic review and meta-analysis	A total number of 46 265 breast cancer patients from 11 studies were included. 10 were independent studies on beta blocker use and 7 were on ACEi/ ARB use	The events of interest were breast cancer survival and DFS for patients at any stage. If breast cancer-specific survival was not available, overall survival and a sensitivity analysis was carried out	The summary HR was estimated by pooling the study-specific estimates with the random effects models	We found a significant improvement in breast cancer specific survival for patients treated with beta blocker drugs at the time of breast cancer diagnosis (SHR: 0.44; 95% CI 0.26–0.73 with I^2^ 78%) was found. There was also a borderline significant improvement in disease free survival for subjects treated with beta-blocker (SHR:0.71, 95% CI 0.19–1.03). No association of ACEi/ARB use with disease free and overall survival was found. The authors concluded that beta blockers improve breast cancer-specific survival and that clinical trials addressing this hypothesis were warranted	N/A
Beta-blocker use and breast cancer outcomes: a meta-analysis	Scott et al. (2024)	Meta-analysis of cohort studies	24 studies included 253,082 women with breast cancer	Breast cancer deaths and breast cancer recurrence	When studies reported risk estimates for beta-blockers used both before and after breast cancer diagnosis for the same cohort, the authors only used risk estimates for post-diagnosis use were taken (to avoid sample overlap), as it is usually the more clinically relevant exposure period. Risk estimates and 95% confidence intervals were transformed onto the log scale. To pool risk estimates into a summary estimate, the inverse variance method with random effects model was used	No statistically significant association between beta-blocker use and both breast cancer death (19 studies, hazard ratio = 0.90, 95% CI 0.78–1.04) and breast cancer recurrence (16 studies, HR = 0.87, 95% CI 0.71–1.08) was found. After removing studies with immortal time bias, the associations were attenuated towards the null hypothesis. No o effect modification for either outcome was found when stratifying by the exposure period or type of b-blocker. There was clear evidence of publication bias for both outcomes	The mean or median follow-up time across the 24 studies ranged from 2.1 to 10.5 years
Perioperative COX-2 and beta-Adrenergic Blockade Improves Metastatic Biomarkers in Breast Cancer Patients in a Phase-II Randomized Trial	Shaashua et al. (2017)	Clinical trial	38 women (age 33–70) diagnosed with stage I–III breast cancer were enrolled from three medical centres in Israel. Drug/placebo was administered for 11 consecutive days, starting 5 days before resection of the primary tumour. Oral BID etodolac (400 mg) was administered throughout the treatment period. Propranolol was administered orally using extended release formulations: 20 mg BID during the 5 days preceding surgery; 80 mg on the morning of surgery and on the evening and morning following surgery; and 20 mg BID thereafter during 5 postoperative days. Identical schedules were used for placebo and medication	Tumour gene expression, tumour molecular biomarkers, inflammatory indicators in the tumour and circulation. Blood sample data during treatment were expressed as a percentage of the average value at no-treatment time points	For tumour transcriptome analyses, the statistical significance of bioinformatic result ratios (Drugs/Placebo) was tested by the student t test	Drug treatment: Reduced mesenchymal polarization Z score -0.43 ± SE 0.09 *p* < 0.001 Decreased epithelial to mesenchymal transition (EMT) Reduced expression of CD14 + monocyte Reduced activity of pro-metastatic/proinflammatory transcription factors Increased expression of genes characteristic of CD19 + B cells Decreased tumour-infiltrating monocytes while increasing tumour-infiltrating B cells	5 post-operative days
Beta blockers and improved progression-free survival in patients with advanced HER2 negative breast cancer: a retrospective analysis of the ROSE/TRIO-012 study	Spera et al. (2017)	Randomised controlled trial (retrospective analysis)	A total of 153 patients (13% of the Intent to treat (ITT) population) received beta-blockers during TRIO-012 trial; 71% of them (N ¼ 108) were randomized to the ramucirumab arm	The impact of beta blockers on cancer outcomes	Retrospectively identified in the ITT population those receiving concomitant treatment with any dose of beta-blocker during the study (either during the active treatment phase and/or within 30 days prior to randomisation) Progression-free survival (PFS) and overall survival were estimated using the Kaplan–Meier method, with different treatment cohorts compared using the Log-Rank test. Cox proportional models were used to estimate the HR) for PFS and OS based on beta-blocker intake	Median PFS in beta-blocker treated patients was longer than in patients who did not receive them (10.3 versus 8.3 months; HR 0.81; 95% CI 0.66–0.99; *P* ¼ 0.038). Patients treated with beta-blocker only after enrolment had even higher median PFS (15.5 versus 8.3 months, *P* < 0.001). In the TNBC subset, median PFS was 13.0 months with beta-blocker, compared to 5.2 months without beta-blocker (HR 0.52; 95% CI 0.34–0.79; *P* ¼ 0.002). Beta-blocker intake was associated with significant improvement in PFS, particularly in patients with TNBC and patients not previously exposed to beta-blockers	Median duration 25.1 months
Evidence of beta-blockers drug repurposing for the treatment of triple negative breast cancer: A systematic review	Spini et al. (2019)	Systematic review of pre-clinical studies	46 preclinical studies and 3 clinical papers included assessing the effect of any molecule with adrenergic activity on triple negative breast cancer (TNBC) models. Clinical studies concerning the exposure to any beta-blockers in TNBC patients were also eligible N = 377 and 800 women (not clear for the third study)	To review evidence collected from preclinical and clinical studies concerning the repurposing of beta-blockers in TNBC	Narrative synthesis	In in vitro studies, propranolol (non-selective beta-blocker) significantly decreased proliferation, migration and invasion of TNBC cells In vivo studies, propranolol inhibited metastasis, angiogenesis and tumour growth Two clinical studies showed a reduced risk of recurrence and metastasis and risk of death	N/A
β-Adrenergic Receptors: New Target in Breast Cancer	Wang et al. (2015)	Systematic review	10 articles were eligible based on: (1) the study design was interventional;(2) the breast cancer patients should be confirmed thepathological type; (3) the usage of Beta-adrenoceptorblockers were evaluated; (4) clinical studies used a control group excluding animal and cell line studies; (5) clinical outcomes should be provided, such as survival, disease-free survival (DFS), tumour recurrence and metastasis	Survival, disease-free survival (DFS), tumour recurrence and metastasis	Hazard ratios	Six suggested that receiving beta- adrenoceptor blockers reduced the risk of breast cancer–specific mortality, and three of them had statistical significance (hazard ratio (HR) = 0.42; 95% CI = 0.18–0.97; *p*= 0.042). Two studies reported that risk of recurrence and distant metastasis (DM) were both significantly reduced. One study demonstrated that the risk of relapse-free survival (RFS) was raised significantly with beta-blockers (beta-blockers) (HR = 0.30; 95% CI = 0.10–0.87; *p* = 0.027). One reported longer disease-free interval (Log Rank (LR) = 6.658; p = 0.011) in beta-blockers users, but there was no significant association between overall survival (OS) and beta-blockers (HR = 0.35; 95% CI = 0.12–1.0; *P* = 0.05) in five studies	Average length of follow up ranged from 1.3 years to 10.5 years
Impact of Beta Blockers on Breast Cancer Incidence and Prognosis	Yang et al. (2023)	Meta-analysis	43 articles	Cancer-specific mortality (CSM), all-cause mortality (ACM),disease-free survival (DFS), overall survival (OS), cancer-specific survival (CSS), progression free survival (PFS), and recurrence	Random effects model if heterogeneity was significant, while the fixed effects model was used if heterogeneity was not significant. Risk ratio and 95% CI were used to assess the effect size. The comparative groups were beta-blocker users versus beta-blocker nonusers. If the RR was not reported, assumed that OR or HR could be considered approximatively equivalent to the RR	beta-blocker increased breast cancer risk (n = 22, RR: 1.169, 95% CI1.063–1.285). We also found beta-blocker were associated with a lower overall survival (OS, n = 19, RR: 1.125, 95% CI 1.078–1.173) and a higher recurrence risk (n = 8, RR: 1.130, 95% CI 1.040–1.227) for breast cancer. beta-blocker were associated with a significantly lower overall survival (n = 3, RR: 2.751, 95% CI 1.213–6.238) and higher recurrence (n = 2, RR: 1.284, 95% CI 1.102–1.497) only in luminal breast cancer while with a higher PFS (n = 2, RR: 0.585, 95% CI 0.343–0.997) in Her2 + breast cancer. No significant differences in terms of CSM (n = 19, RR: 1.009, 95% CI 0.947–1.077), PFS (n = 4, RR: 0.932, 95% CI 0.616–1.305), and DFS (n = 2, RR: 0.776, 95% CI 0.512–1.176) were observed	36–124 months
Propranolol Attenuates Surgical Stress-Induced Elevation of the Regulatory T Cell Response in Patients Undergoing Radical Mastectomy	Zhou et al. (2016)	Clinical trial	154 women between the ages of 25 and 65 who underwent a modified radical mastectomy for the treatment of primary breast cancer Patients were randomly assigned to control, propranolol, parecoxib, and propranolol plus parecoxib groups. In the propranolol group, patients were treated with oral propranolol (20 mg three times daily) from the day of surgery until the third postoperative day. In the parecoxib group, patients received i.v. parecoxib (40 mg once daily) from the day of surgery until the second postoperative day. In the propranolol plus parecoxib group, patients received propranolol and parecoxib by the oral and IV routes, respectively	Level of CD4 + CD25 + FOXP3 + Tregs in the peripheral blood of breast cancer patients	Continuous variables were defined as means ± SD if they were normally distributed; otherwise, median values and interquartile ranges (25th–75th percentile) were represented. ANOVA, two-way ANOVA, paired or unpaired t tests, Mann–Whitney tests, Wilcox signed ranks tests, Fisher’s exact tests, and McNemar tests, were used as appropriate. The p values < 0.05 were considered statistically significant in all studies, and all p values were two tailed	Propranolol administration alone was able to diminish the increased Treg level/ activity observed in response to mastectomy	7 days post-op

Shaashua et al. (2017) and Haldar et al (2018) conducted a small clinical trial including 38 patients with breast cancer. There were 19 patients in the control arm and 19 in the intervention arm which also included taking twice-daily etodolac (a COX-2 inhibitor) throughout the treatment period. Propranolol was administered orally using extended-release formulations: 20 mg twice-daily during the 5 days preceding surgical resection of the tumour; 80 mg on the morning of surgery and on the evening and morning following surgery; and 20 mg twice-daily thereafter during 5 postoperative days.

Shaashua et al. (2017) found that perioperative administration of propranolol led to (i) decreased epithelial-to-mesenchymal transition (EMT), (ii) reduced expression of CD14 + monocytes and (iii) increased expression of genes characteristic of CD19 + B cells, each of which represent surrogate indicators of patient prognosis.

Halder et al. (2018) concluded that the drug treatment significantly reduced serum levels of pro-inflammatory cytokines, which are often associated with poorer cancer outcomes (Trompet et al. 2009). Additionally, the treatment decreased the activity of multiple inflammation-related transcription factors, highlighting a potential pathway for improved prognosis (Haldar et al. 2018). In the excised tumour tissue, those in the treatment arm showed a notable reduction in Ki-67 expression, a key marker of cell proliferation that correlates with tumour aggressiveness and likelihood of recurrence.

Zhou et al. (2016) investigated the effect of the perioperative use of propranolol or parecoxib on the number and activity of peripheral T-regulator cells (Tregs) in 154 breast cancer patients who underwent a radical mastectomy. The percentage of Tregs in the propranolol group and the propranolol plus parecoxib group did not increase at 7 days post-op compared with the control group. The combination of propranolol and parecoxib did not exhibit higher efficacy than propranolol alone, indicating that these two drugs may not be synergistic.

The main limitations to these studies include small sample sizes and lack of long-term follow-up to assess impacts of perioperative COX-2 and beta-adrenergic inhibition on clinical rather than surrogate outcomes such as tumour biomarkers and gene expression in early-stage breast cancer.

A phase 2 triple-blind placebo-controlled RCT (Hiller et al. 2020), including 60 female patients aged 18–80 years with early-stage primary breast cancer, found that propranolol reduced EMT-related and inflammatory signalling within tumours and enhanced immune cell recruitment to breast cancer; biomarkers associated with metastatic potential. The main limitations of this trial were that only 30 patients were recruited per randomised intervention and a surrogate primary endpoint was investigated (the quantitative change in tumour tissue pro-metastatic gene expression), which makes it difficult to draw clinically meaningful conclusions. In addition, four patients were also lost to follow-up due to withdrawal prior to commencing medication and non- completion of adequate pre-operative dosing.

Finally, the TRIO-012 trial included 153 patients with advanced HER2 negative breast cancer who received beta-blockers (n = 13, propranolol) with 71% of total patients receiving them as an adjunct to ramucirumab (Spera et al. 2017). The authors concluded that beta-blockers were associated with improved progression free survival, particularly for the subgroup of patients with triple-negative disease. Conversely, there was no significant difference in the overall survival for patients who received beta-blockers versus those who did not, regardless of timing of beta-blocker use and breast cancer subtype.

In a more recent meta-analysis of 24 studies (Scott et al. 2024), pooled results showed no evidence of an association between beta-blocker use and either breast cancer death (19 studies, hazard ratio = 0.90, 95% CI 0.78–1.04) or breast cancer recurrence (16 studies, HR = 0.87, 95% CI 0.71–1.08). Another meta-analysis reported similar results from 17 observational studies (Li et al. 2020). There was no significant association between beta-blocker use and breast cancer recurrence (RR = 0.85, 95% CI 0.68–1.07, *P* = 0.17), breast cancer related deaths (RR = 0.83, 95% CI 0.65–1.06, *P* = 0.14), or all-cause deaths (RR = 1.01, 95% CI 0.91–1.11, *P* = 0.91) (Li et al. 2020). Study characteristics such as sample size, follow-up durations or adjustment of menopausal status did not significantly affect the results.

Conversely, Raimondi et al. (2016) performed a meta-analysis that included more than 46,000 patients from 11 breast cancer studies (non-RCTs, primarily cohort studies) and found a significant improvement in breast cancer-specific survival for patients treated with beta-blockers. Weberpals et al. (2016) also found evidence of improved overall and cancer-specific survival with these drugs in their systematic review and meta-analysis that included more than 88,000 patients with ovarian, colorectal, lung, prostate and breast tumours from 30 studies. However, this improvement was not confirmed when eleven studies were excluded due to being deemed prone to immortal time bias, not providing sufficient information or having no exposure definition to ensure that there was no immortal time bias.

A meta-analysis of eight European patient cohorts found that beta-blocker treatment before or after the diagnosis of breast cancer was not associated with a decreased risk of breast cancer or all-cause mortality (Cardwell et al. 2016). However, other reviews (Powe et al. 2010), including one specifically looking at triple negative breast cancer (Spini et al. 2019), demonstrated the opposite results, with patients treated with beta-blockers showing a reduction in metastasis development, tumour recurrence and a longer disease-free survival. Moreover, beta-blockers led to a decreased risk of cancer-specific mortality and all-cause mortality when used *after* diagnosis, as demonstrated in another systematic review (Zhou et al. 2016).

### Other cancers

Table 2 shows the characteristics of reviews and trials that evaluated beta-blockers and other cancer types included in this analysis. Two of these were randomised controlled trials that focused on multiple myeloma (Knight et al. 2020) and ovarian cancer (Heitz et al. 2013).

**Table 2:** Characteristics and findings of studies and reviews that investigated the use of beta-blockers on all cancers except breast cancer (n = 14)

Title	Author, year	Study type	Cancer type	Population characteristics	Cancer outcomes	Statistical analysis	Results/key findings	Length of follow-up
Beta-adrenergic receptor blockers and hepatocellular carcinoma survival: a systemic review and meta-analysis	Chang and Lee (2023)	Systematic review and meta-analysis	Hepato-cellular carcinoma	Four studies involving 7252 patients with HCC	Primary outcome was to assess the association between beta-blocker use and overall survival (OS) and cancer-specific survival (CSS) in patients with HCC	Random-effects model to calculate the pooled Hazard rations (HRs and their 95% Confidence intervals (CI)	The pooled results indicated that beta-blockers were associated with better OS in patients with HCC (HR = 0.69, 95% CI = 0.54–0.88, *P* = 0.0031). There was no significant heterogeneity among the three studies (*I*^*2*^ = 41%, 95% CI = 0.0%- 82.2%; *Q* = 6.42, *P* = 0.18)	N/A
Meta-analysis of the effects of beta blocker on survival time in cancer patients	Choi et al. (2014)	Meta-analysis	Breast, ovarian, prostate, melanoma and mixed cancers	18 studies were included based on data obtained from 20,898 subjects	Primary outcome was OS. Another point of interest was disease-free survival (DFS)	Effect sizes (assessed by HRs) were heterogeneous, and random-effects models were used in the analyses	Beta blocker use was associated with improved OS (HR 0.79; 95% CI 0.67–0.93; *p* = 0.004) and DFS (HR 0.69; 95% CI 0.53–0.91; *p* = 0.009). Although statistically not significant, the effect size was greater in patients with low-stage cancer or cancer treated primarily with surgery than in patients with high-stage cancer or cancer treated primarily without surgery (HR 0.60 vs. 0.78, and 0.60 vs. 0.80, respectively). The studies using nonselective beta blockers (n = 2) showed that there was no overall effect on OS (HR 0.52, 95% CI 0.09–3.04)	17– 122 months
Effect of beta-blockers on survival of lung cancer patients: a systematic review and meta-analysis	Coelho et al. (2020)	Systematic review and meta-analysis	Lung cancer	Of 920 studies, seven (all retrospective or observational studies—six cohort and one case–control), including 7448 patients, met the inclusion criteria	OS	HR and 95% CIs for OS were estimated using a random-effects model	Beta-blocker users with lung cancer has no increased OS compared to non-users at random effect model (HR = 1.00; 95% CI = 0.91–1.10; I2 = 45%)	N/A
Impact of beta blocker medication in patients with platinum sensitive recurrent ovarian cancer-a combined analysis of 2 prospective multicentre trials by the AGO Study Group, NCIC-CTG and EORTC-GCG	Heitz et al. (2013)	Clinical trial	Ovarian cancer	Included patients received treatment within two prospective clinical trials: AGO-OVAR 2.4 phase I trial (carboplatin/ gemcitabine; N = 25, protocol AGO-OVAR 2.4) and AGO led intergroup phase III trial (carboplatin vs carboplatin/gemcitabine; N = 356, protocol AGO-OVAR 2.5, EORTC-GCG, NCIC CTG). Concurrent	Progression-free survival (PFS)—defined as the time from the date of randomization to the date of disease progression or death from any cause, and OS	Independence of continuous variables was assessed using Student’s t-test. Comparison of two or more groups of binary variables used the χ2-test. Multivariate analyses were performed using the Cox-regression model. The Kaplan–Meier method including a log-rank test was used to show unadjusted survival differences	349 (91.6%) patients had progressive disease and 267 (70.1%) had died. No difference in median progression-free survival (PFS) (7.79 vs. 7.62 months (p = 0.95)) and OS (21.2 vs 17.3 months (*p* = 0.18)) was recorded for patients treated with and without beta blockers. medication was documented after every cycle with thorough monitoring	Median follow-up of 17 months
Propranolol inhibits molecular risk markers in HCT recipients: a phase 2 randomized controlled biomarker trial,	Knight et al. (2020)	Randomised controlled trial	Multiple myeloma	25 patients aged 18–75 years receiving an autologous hematopoietic cell transplant (HCT) for multiple myeloma Intervention (n = 12), Control (n = 13) Propranolol 20 mg was taken twice daily a week before HCT, then 40 mg twice daily until day 28 post-transplant	The primary objective was to assess whether beta-blocker administration to individuals undergoing HCT reduces (1) Conserved Transcriptional Response to Adversity (CTRA) gene expression (an a priori–defined gene set and known risk factor for poor HCT outcomes and (2) myeloid lineage bias in the recovering PBMC pool (which is also prognostic of poor outcomes). Secondary objectives included assessment of safety (adverse event [AE] rates) and quantification of differences in hematopoietic engraftment and infection rates	Intention-to-treat (ITT) analyses controlling for demographic characteristics, high-risk disease (International Myeloma Working Group risk score), and tumour stage tested effects on CRTA genes	Propranolol-treated patients showed decreases from baseline to HCT day -two and day + 28 for CTRA gene expression (0.017). Propranolol-treated patients showed decrease of CD16- classical monocyte activation. (*p* = 0.005) Propranolol-treated patients showed relative upregulation of CD34 + cell-associated gene transcripts (*p* = 0.011) and relative downregulation of myeloid progenitor-containing CD33 + cell-associated gene transcripts (*p* < 0.001)	Longest follow-up at six weeks
Beta-blocker and survival in patients with lung cancer: A meta-analysis	Lei et al. (2021)	Meta-analysis	Lung cancer	10 studies included in the meta-analysis and quantitative analysis	Association between beta-blocker use and overall survival of lung cancer	Adjusted HRs. A random effect model was used to pool the results	Beta-blocker use was not associated with significantly affected OS in lung cancer (adjusted HR = 1.02, 95% CI 0.98 to 1.06, *p* = 0.33), with moderate heterogeneity (I2 = 29%)	Mean follow-up durations varied from 1.6 to 6.5 years
Common medications and survival in women with ovarian cancer: A systematic review and meta-analysis	Majidi et al. (2020)	Systematic review and meta-analysis	Ovarian cancer	36 studies included Study populations were women with invasive ovarian (or fallopian tube or primary peritoneal) cancer. Survival of users and non-users were compared	Endpoints were ovarian cancer-specific survival, (OVS) or any cause (OS), or progression-free survival, PFS)	Inverse variance method with random-effects models to generate pooled hazard ratios (pHR). Where necessary, used the reported P-value to estimate the relevant confidence limits	The meta-analysis of the ITB-free studies suggested improved survival in statin users compared to non-users (pooled HR: 0.76, 95% CI 0.68–0.85), but no overall survival benefit associated with use of beta-blockers (pooled HR: 1.07, 95% CI 0.96–1.21)	N/A
Beta-blockers and glioma: a systematic review of preclinical studies and clinical results	Tewarie et al. (2021)	Systematic review of pre-clinical studies	Glioma	Ten preclinical studies and one clinical study were included	Association between beta-blockers and survival in glioma patients	Narrative synthesis	The one clinical study did not find an association between beta-blockers and survival in glioma patients. Although preclinical studies provide scarce evidence for the use of beta-blockers in glioma, they identified potential pathways for targeting glioma	N/A
The association between beta-blockers use and prostate cancer mortality: A mini systematic review and meta-analysis	Uleri et al. (2024)	A mini systematic review and meta-analysis	Prostate cancer	10 studies met inclusion criteria and a total of 74,970 patients were included: 26,674 beta‐blocker users and 48,326 non-users	Primary outcome was prostate cancer mortality in beta‐blocker users versus non-users. OS was studied as a secondary endpoint	The inverse variance method with adjusted hazard ratios (HRs) and 95% confidence intervals (CIs) derived from the Cox proportional hazard model were used to determine the association between beta‐blockers use and survival outcomes	There was no statistically significant association between beta‐blocker exposure and prostate cancer mortality ( HR 0.97; 95% CI 0.87–1.09; *p* = 0.61). Similar results for analysis restricted to studies including only patients with advanced disease (HR 0.92; 95% CI 0.80–1.06; *p* = 0.24). Similarly, no association with overall survival (HR 1.02; 95% CI 0.94–1.10; p = 0.64)	30–134 months
Beta adrenergic blockade and clinical outcomes in patients with colorectal cancer: A systematic review and meta-analysis	Wang et al. (2022)	Systematic review and meta-analysis	Colorectal cancer	14 studies involving 85,993 patients included	Primary endpoint was cancer-specific mortality, defined as the occurrence of colorectal cancer-related death The secondary endpoints were overall 1-year mortality, PFS and OS according to beta-blocker use	HRs and 95% CIs were extracted and summarized for each study to compare colorectal cancer patients with or without beta blockade. Heterogeneity was assessed using the χ2test and quantified (values > 50% indicated moderate-to-high heterogeneity). Pooled effect was calculated using the random effects model if moderate-to-high heterogeneity existed and fixed effects model if not	The use of beta blockade was associated with improvements in cancer-specific mortality (N = 59,621; HR 0.87; 95% CI, 0.76–0.99; *P* = 0.04) and overall, 1-year mortality (N = 37,442; HR 0.54; 95% CI, 0.43–0.67; *P* < 0.00001), while there was no significant difference in overall survival (N = 37,975; HR 0.95; 95% CI, 0.85–1.05; *P* = 0.28). In patients with stage IV colorectal cancer, the use of beta blockade was significantly associated with improved progression-free survival (N = 749; HR 0.76; 95% CI, 0.62–0.92; *P* = 0.005)	1.42 to 79.2 months
Beta blockers and cancer prognosis—The role of immortal time bias: A systematic review and meta-analysis,	Weberpals et al. (2016)	Systematic review and meta-analysis	Colorectal, breast, ovarian, prostate, non-small cell lung cancer, melanoma, acute myeloid leukaemia and mixed cancer sites	30 eligible studies including 88,026r patients in total	Beta-blocker users had a significantly better overall (HR 0.88, 95% CI 0.79–0.97) and cancer-specific (HR 0.75, 95% CI 0.64–0.88) survival. Excluding the studies considered prone to immortal time bias (ITB) resulted in HRs (95% CIs) of 1.00 (0.93–1.07) and 0.90 (0.83–0.98), respectively. Analyses of cancer site and beta-blocker type did not show beneficial associations besides OS among melanoma patients	Secondary analyses of the association of beta blockers with cancer prognosis stratified for each cancer site and beta-blocker type. Random effects model and pooled the log (HR) across studies and weighting each study by its standard error	Beta-blocker users had a significantly better OS (HR 0.88, 95% CI 0.79–0.97) and cancer-specific (HR 0.75, 95% CI 0.64–0.88) survival. Excluding the studies deemed to be prone to ITB resulted in HRs (95% CIs) of 1.00 (0.93–1.07) and 0.90 (0.83–0.98), respectively. Analyses on cancer site and beta-blocker type did not show beneficial associations besides overall survival among melanoma patients. However, melanoma-specific survival was not improved	0.1–10.5 years
Post-Diagnostic Beta Blocker Use and Prognosis of Ovarian Cancer: A Systematic Review and Meta-Analysis of 11 Cohort Studies With 20,274 Patients	Wen et al. (2021)	Systematic review and meta-analysis	Ovarian Cancer	11 cohort studies with 20,274 patients	Cancer mortality, all-cause mortality, overall survival, progression-free survival	Random-effects model was applied for summarizing HRs	The summary HRs did not reveal any statistically significant associations between post-diagnostic beta blocker use and ovarian cancer prognosis characteristics, such as total mortality (HR = 1.08, 95% CI = 0.92–1.27, I2 = 76.5%, n = 9), cancer-specific mortality (HR = 1.22, 95% CI = 0.89–1.67, I 2 = 88.1%, n = 3), and progression-free survival (HR = 0.88, 95% CI = 0.75–1.05, I2 = 0, n = 4)	Median follow-up duration between 17 and 91 months
Effect of beta-blockers on cancer recurrence and survival: a meta-analysis of epidemiological and perioperative studies	Yap et al. (2018)	A meta-analysis of epidemiological and peri-operative studies	Breast, colorectal, endometrial, head and neck, lung, renal, prostate, pancreas, melanoma	27 studies included. Nine used non-selective beta blockers, ten were perioperative studies	Association between beta-blocker use and cancer recurrence (CR), DFS, and OS	Transformed reported HR estimates into log hazard ratios. Extracted 95% confidence intervals were transformed to standard errors. The generic inverse variance method with random-effects model was used for pooled estimates	Beta-blocker use had no effect on CR. Within subgroups of cancer, melanoma was associated with improved DFS (HR 0.03, 95% CI 0.01–0.17) and OS (HR 0.04, 95% CI 0.00–0.38), while endometrial cancer had an associated reduction in DFS (HR 1.40, 95% CI 1.10–1.80) and OS (HR 1.50, 95% CI 1.12–2.00). There was also reduced OS seen with head and neck and prostate cancer. Non-selective beta-blocker use was associated with improved DFS and OS in ovarian cancer, improved DFS in melanoma, but reduced OS in lung cancer	N/A
β-Blocker use and mortality in cancer patients: systematic review and meta-analysis of observational studies	Zhong et al. (2016)	Systematic Review and Meta-analysis of observational studies	All cancers	24 studies included in the meta-analysis with 2286 screened	Assess the relationship between post-diagnostic and pre-diagnostic β-blocker use and the survival of cancer patients for both all-cause mortality and cancer-specific mortality. In addition, a dose–response analysis was carried out to further evaluate the potential dose–response relationship	Random-effects model where the restricted maximum likelihood estimator was used to evaluate the inter-study heterogeneity	The overall results showed that patients who used beta- blockers after diagnosis had an HR of 0.89 (95% CI 0.81–0.98) for all-cause mortality compared with non-users. Those who used beta- blockers after diagnosis (vs. nonusers) had an HR of 0.89 (95% CI 0.79–0.99) for cancer-specific mortality. Pre-diagnostic use of beta- blockers showed no beneficial effect on all-cause mortality or cancer-specific mortality. Stratifying by cancer type, only breast cancer patients who used beta-blockers after diagnosis had a prolonged OS	3–10 years

Knight et al. (2020) conducted a trial with 25 patients between 18–75 years receiving an autologous hematopoietic cell transplant (HCT) for multiple myeloma, randomised to intervention (n = 12) and control (n = 13) arms. The intervention arm involved propranolol 20 mg was taken twice daily a week before HCT, then 40 mg twice daily until day 28 post-transplant. Propranolol-treated patients showed significant reductions in expression of CTRA (Conserved Transcriptional Response to Adversity– a cell cycle response regulator) indicator genes (primary outcome). Relapse and reduced disease-free survival are associated with heightened beta-adrenergic signalling and CTRA biology (Knight et al. 2015) and may have promise as future clinical study biomarker end points. In addition, significant reductions in myeloid lineage bias and proinflammatory tissue factor activity, and significant increases in innate antiviral and lymphoid lineage-related tissue factor activity (secondary outcomes) during hematologic recovery were seen. The study suggests that propranolol may have capacity to inhibit the sympathetic nervous system-regulated, bone marrow–derived adverse gene expression profile previously associated with poor HCT outcomes (Knight et al. 2015), shift cell differentiation away from a myeloid bias, and promote engraftment, yet is ultimately limited by sample size.

On the other hand, Heitz et al. (2013) recruited 38 patients with ovarian cancer, who received beta-blockers as a co-medication to chemotherapy (carboplatin/gemcitabine) and found that response rates to chemotherapy were not different between patients treated with beta-blockers and those who were not. After a median follow-up of 17 months, there was no difference in median progression-free survival (7.79 vs. 7.62 months, *p* = 0.95) and overall survival (21.2 vs. 17.3 months, *p* = 0.18) recorded for patients treated with and without a beta blocker.

Similarly, in a systematic review and meta-analyses of 36 studies on ovarian cancer, no overall survival benefit associated with use of metformin, beta-blockers, aspirin or NSAIDs were found (Majidi et al. 2020). The pooled result of two studies did, however, suggest a possible association between *perioperative* beta-blocker use and improved survival (Al-Niaimi et al. 2016; Heitz et al. 2013). Nevertheless, Yap et al. (2018) found that there were no statistically significant associations overall between perioperative beta blocker use and ovarian prognosis, such as total mortality (HR = 1.08, 95% CI = 0.92–1.27, I^2^ = 76.5%, n = 9), cancer-specific mortality (HR = 1.22, 95% CI = 0.89–1.67, I^2^ = 88.1%, n = 3), and progression-free survival (HR = 0.88, 95% CI = 0.75–1.05, I^2^ = 0, n = 4).

Further similar results have been echoed in two meta-analyses on beta-blocker use in lung cancer, which both reported no significant association between beta-blocker use and overall survival (Coelho et al. 2020; Lei et al. 2021) and prostate cancer (Uleri et al. 2024).

Zhong et al. (2016) conducted a meta-analysis of 24 studies on all cancers assessing the relationship between pre- and post-diagnostic beta-blocker use and the survival of cancer patients. The overall results showed that patients who used beta-blockers after their cancer diagnosis had lower all-cause mortality and cancer mortality compared with non-users with hazard ratios of 0.89. Pre-diagnostic use of beta-blockers showed no beneficial effect on all-cause mortality or cancer-specific mortality. Stratifying by cancer type, only breast cancer patients who used beta-blockers *after* diagnosis had a prolonged overall survival.

## Quality assessment and limitations

The Cochrane risk of bias tool (Higgins et al. 2011) was used to assess risk of bias for the 7 included RCTs (Table 3). The studies demonstrated varying quality in assessing the role of propranolol. Most studies, such as those by Haldar et al. (2018) and Spera et al. (2017), used rigorous methodologies with computerised randomisation, blinding, and intention-to-treat analyses, effectively minimising selection, attrition, and reporting biases. Hiller et al. (2020) further strengthened validity using a triple-blind design and clearly defined endpoints. Conversely, the studies by Heitz et al. (2013) and Knight et al. (2020) lacked blinding, increasing their susceptibility to bias and suffered from small sample sizes (< 30) and therefore, lack of statistical power. Zhou et al. (2016) employed proper allocation concealment but failed to blind outcome assessors, raising concerns about performance bias. Overall, while several studies applied robust quality controls, inconsistencies in blinding and outcome assessment highlight the need for more uniformly rigorous research.

**Table 3:** Cochrane Risk of Bias assessment for 7 included RCTs

	Random sequence generation (selection bias)	Allocation concealment (selection bias)	Blinding of participants and personnel (performance bias)	Incomplete outcome data (attrition bias)	Blinding of outcome assessment (detection bias)	Selective reporting (reporting bias)
Haldar et al. (2018)	L	U	L	H	L	L
Heitz et al. (2013)	L	L	U	H	U	L
Hiller et al. (2020)	L	L	L	H	L	L
Knight et al. (2020)	L	L	U	H	U	L
Shaashua et al. (2017)	L	L	L	H	L	L
Spera et al. (2017)	L	L	L	L	L	L
Zhou et al. (2016)	L	L	U	L	U	L

*L* = low risk of bias, *U* = unclear risk of bias, *H* = high risk of bias

Further limitations also hinder definitive conclusions about the role of propranolol in cancer treatment by this review. First, there were very few clinical trials, and most were observational, which makes it difficult to rule out confounding factors such as patients' underlying health conditions or lifestyle choices. Additionally, the heterogeneity of the studies in terms of patient populations, cancer types, and treatment regimens makes it difficult to draw generalisable conclusions and conduct a meta-analysis.

## Discussion

This systematic review set out to evaluate the potential role of propranolol, a non-selective beta-blocker, in cancer treatment, with a specific focus on breast cancer. A key distinction of this review is the emphasis on propranolol specifically. Further, while recent systematic reviews evaluated beta-blockers more broadly, our review interrogates both systematic reviews and new RCT data that isolate propranolol. An earlier meta-analysis (Caparica et al. 2021) emphasised general class effects in early-stage breast cancer and showed mixed results without demonstrating a consistent benefit. Another study by Scott et al. (2024) found no clear association between beta-blocker use (across both selective and non-selective agents) and breast cancer recurrence or mortality even after stratifying by exposure period or beta-blocker subtype in a study through August 2023. We include several recent trials not yet synthesised in earlier reviews and discuss propranolol’s unique non-selective profile and mechanistic rationale in cancer biology. Additionally, our review identifies dosage- and timing-specific signals, whereas evidence remains inconclusive for adjunctive use with chemotherapy or radiotherapy. By honing in on propranolol’s pharmacologic profile and targeted clinical use, this review delivers a mechanistically informed synthesis to guide future trial design, offering further clarity that prior broad-spectrum beta-blocker reviews could not.

Our analysis of the epidemiological and clinical trial literature suggests that propranolol may have a promising role in certain aspects of cancer treatment. However, its overall effectiveness remains inconclusive and may be dependent on context, such as the potential significance of the timing of administration in relation to each of surgery, chemotherapy, and radiotherapy. We also explore some underlying biological mechanisms that may explain the effects of propranolol on cancer cells.

The epidemiological data on beta-blockers and cancer incidence or survival appear to show mixed outcomes, with some studies indicating a potential protective effect (Raimondi et al. 2016), while others suggest minimal to no benefit (Caparica et al. 2021; Kim et al. 2017; Scott et al. 2024; Yap et al. 2018). The potential role of beta-blockers as adjuvant therapy in cancer treatment is supported by preclinical studies that suggest that propranolol may slow tumour growth, reduce metastasis, and improve the overall response to cancer therapies (Bucsek et al. 2017; Duckett et al. 2020; Kokolus et al. 2017). Findings from the RCTs included in this review suggest some mechanisms by which propranolol acts to reduce cancer. Shaashua et al. (2017) found that EMT– a process that allows epithelial cells to acquire migratory and invasive properties– was reduced after perioperative administration of a beta-blocker, which makes tumours less capable of invading surrounding tissues and metastasising to distant sites, which is crucial for improving patient prognosis (Ribatti et al. 2020). In addition, reduced expression of CD14 + monocytes, which are typically involved in inflammatory responses within the tumour microenvironment, suggest reduction in tumour growth, decreased progression and metastasis (Gustafson et al. 2015). CD19 + B cells are crucial for the adaptive immune response. Their increased presence and activity can enhance the body's ability to recognise and attack tumour cells, potentially improving patient outcomes. Halder et al. (2018) commented on their findings of higher levels of the transcription factors SP1 and AHR which are theorised to play a role in several pathways, including invasion, Insulin-like Growth Factor (IGF) signalling, inflammation, DNA repair and kynurenine metabolism. These factors contribute to enhanced tumour regression and are associated with good metastasis-free survival (Vacher et al. 2018).

However, these findings are not universally observed, and the inconsistency across studies could be due to variations in study design, patient characteristics, and the timing of beta-blocker administration.

One key factor that emerges from the literature is the timing of propranolol administration in relation to other cancer treatments: Studies suggest that beta-blockers may have a more pronounced effect when used perioperatively, in conjunction with surgical treatment (Al-Niaimi et al. 2016; Haider et al. 2020; Haldar et al. 2018; Huettner et al. 2020; Ricon-Becker et al. 2023; Shaashua et al. 2017; Yap et al. 2018). The perioperative period is a time of heightened stress and inflammation, which is known to influence cancer progression (Hiller et al. 2018). By mitigating the effects of stress hormones such as norepinephrine, propranolol could therefore reduce tumour cell proliferation and metastasis during this vulnerable window.

Additionally, the use of propranolol as an adjunct to other cancer therapies, such as chemotherapy or radiotherapy, has gathered interest due to the potential synergistic effects (Carnet Le Provost et al. 2023). Specifically, there is some evidence that propranolol could enhance the effects of radiotherapy by improving the tumour’s sensitivity and exert a T cell-dependent immune response that effectively slows tumour growth (Duckett et al. 2020; Liao et al. 2018). Moreover, propranolol decreases the expression of pro-metastatic, pro-inflammatory and pro-angiogenic genes such as EGFR, COX-2 and VEGF, respectively, thus impairing cell viability and inducing apoptosis (Duckett et al. 2020; Wolter et al. 2014). In preclinical models, beta-blockade has been shown to decrease hypoxia in tumours, a condition that often limits the effectiveness of radiotherapy (Barathova et al. 2020). By improving blood flow and oxygenation, propranolol may make tumours more susceptible to radiation-induced cell death.

Moreover, the cytotoxic effects of conventional chemotherapeutic agents such as platinum salts, anthracyclines, 5-fluorouracil, mitotic spindle poisons (such as taxanes or vincristine) and topoisomerase inhibitors increased in the presence of β-blocking agents including propranolol (Pasquier et al. 2011). Importantly, propranolol was found to decrease the expression of programmed death receptor-1 (PD-1) in tumours and to increase CD8 + T cell infiltration within the tumour microenvironment, thus enhancing the efficacy of immune checkpoint blockade (Bucsek et al. 2017; Yan et al. 2022). Nevertheless, extensive clinical evidence supporting the combination of beta-blockers with chemotherapy or radiotherapy is still limited.

## Conclusion

In conclusion, while there is some evidence indicating that propranolol may improve cancer outcomes, the timing of administration and adjunctive cancer treatment it is paired with, appear to be key factors in determining its effectiveness. The perioperative period may represent a critical window for beta-blockade to exert its beneficial effects, potentially reducing the risk of recurrence. However, the power of its use in conjunction with chemotherapy or radiotherapy is less clear, with mixed results across studies.

Future clinical trials are needed to address these uncertainties, ideally focusing on specific cancer types, patient populations, and treatment regimens to better define the role of propranolol as an adjunctive therapy. Investigations into the molecular and immunological mechanisms by which beta-blockers exert their effects will be crucial for optimising treatment strategies and identifying the patients who are most likely to benefit from beta-blockade in cancer therapy.

## Electronic supplementary material

Below is the link to the electronic supplementary material.

Supplementary Material 1

## Data Availability

All data analysed during this review are included in the published articles cited in the reference list.

## References

[CR1] Al-Niaimi A, Dickson EL, Albertin C, Karnowski J, Niemi C, Spencer R, Shahzad MMK, Uppal S, Saha S, Rice L, Nally AM (2016) The impact of perioperative β blocker use on patient outcomes after primary cytoreductive surgery in high-grade epithelial ovarian carcinoma. Gynecol Oncol 143(3):521–525. 10.1016/J.YGYNO.2016.09.019 27693123 10.1016/j.ygyno.2016.09.019

[CR2] Ashmore J, Olsen H, Sørensen N, Thrasivoulou C, Ahmed A (2019) Wnts control membrane potential in mammalian cancer cells. J Physiol 597(24):5899–5914. 10.1113/JP278661 31650562 10.1113/JP278661

[CR3] Barathova M, Grossmannova K, Belvoncikova P, Kubasova V, Simko V, Skubla R, Csaderova L, Pastorek J (2020) Impairment of hypoxia-induced CA IX by beta-blocker propranolol—impact on progression and metastatic potential of colorectal cancer cells. Int J Mol Sci 21(22):8760. 10.3390/IJMS21228760 33228233 10.3390/ijms21228760 PMC7699498

[CR5] Berridge MJ, Lipp P, Bootman MD (2000) The versatility and universality of calcium signalling. Nat Rev Mol Cell Biol 1(1):11–21. 10.1038/35036035 11413485 10.1038/35036035

[CR6] Botteri E, Munzone E, Rotmensz N, Cipolla C, De Giorgi V, Santillo B, Zanelotti A, Adamoli L, Colleoni M, Viale G, Goldhirsch A, Gandini S (2013) Therapeutic effect of β-blockers in triple-negative breast cancer postmenopausal women. Breast Cancer Res Treat 140(3):567–575. 10.1007/S10549-013-2654-3 23912960 10.1007/s10549-013-2654-3

[CR7] Bucsek MJ, Qiao G, MacDonald CR, Giridharan T, Evans L, Niedzwecki B, Liu H, Kokolus KM, Eng JWL, Messmer MN, Attwood K, Abrams SI, Hylander BL, Repasky EA (2017) β-Adrenergic signaling in mice housed at standard temperatures suppresses an effector phenotype in CD8+ T cells and undermines checkpoint inhibitor therapy. Can Res 77(20):5639–5651. 10.1158/0008-5472.CAN-17-0546 10.1158/0008-5472.CAN-17-0546 PMC5645237 28819022

[CR8] Caparica R, Bruzzone M, Agostinetto E, De Angelis C, Fêde Â, Ceppi M, de Azambuja E (2021) Beta-blockers in early-stage breast cancer: a systematic review and meta-analysis. ESMO Open. 10.1016/j.esmoop.2021.100066 33639601 10.1016/j.esmoop.2021.100066 PMC7921512

[CR9] Cardwell CR, Pottegard A, Vaes E, Garmo H, Murray LJ, Brown C, Vissers PAJ, O’Rorke M, Visvanathan K, Cronin-Fenton D, De Schutter H, Lambe M, Powe DG, van Herk-Sukel MPP, Gavin A, Friis S, Sharp L, Bennett K (2016) Propranolol and survival from breast cancer: a pooled analysis of European breast cancer cohorts. Breast Cancer Res: BCR 18(1):119. 10.1186/s13058-016-0782-5 10.1186/s13058-016-0782-5 PMC5133766 27906047

[CR10] Carnet Le Provost K, Kepp O, Kroemer G, Bezu L (2023) Trial watch: beta-blockers in cancer therapy. Oncoimmunology 12(1):2284486. 10.1080/2162402X.2023.2284486 38126031 10.1080/2162402X.2023.2284486 PMC10732641

[CR11] Cavalu S, Saber S, Amer AE, Hamad RS, Abdel-Reheim MA, Elmorsy EA, Abdelhamid AM (2024) The multifaceted role of beta-blockers in overcoming cancer progression and drug resistance: Extending beyond cardiovascular disorders. FASEB J 38(13):e23813. 10.1096/FJ.202400725RR38976162 10.1096/fj.202400725RR

[CR12] Chang H, Lee SH (2023) Beta-adrenergic receptor blockers and hepatocellular carcinoma survival: a systemic review and meta-analysis. Clin Exp Med 23(3):853–858. 10.1007/s10238-022-00842-z 35737170 10.1007/s10238-022-00842-z

[CR13] Childers WK, Hollenbeak CS, Cheriyath P (2015) β-Blockers reduce breast cancer recurrence and breast cancer death: a meta-analysis. Clin Breast Cancer 15(6):426–431. 10.1016/j.clbc.2015.07.001 26516037 10.1016/j.clbc.2015.07.001

[CR14] Choi CH, Song T, Kim TH, Choi JK, Park JY, Yoon A, Lee YY, Kim TJ, Bae DS, Lee JW, Kim BG (2014) Meta-analysis of the effects of beta blocker on survival time in cancer patients. J Cancer Res Clin Oncol 140(7):1179–1188. 10.1007/s00432-014-1658-7 24671228 10.1007/s00432-014-1658-7 PMC11823737

[CR15] Coelho M, Squizzato A, Cassina N, Marino F, Ribeiro LV, Cosentino M (2020) Effect of beta-blockers on survival of lung cancer patients: a systematic review and meta-analysis. Eur J Cancer Prevent: off J Eur Cancer Prevent Org (ECP) 29(4):306–314. 10.1097/CEJ.0000000000000544 10.1097/CEJ.0000000000000544 31609808

[CR16] Cole SW, Sood AK (2011) Molecular Pathways: Beta-adrenergic signaling in cancer. Clin Cancer Res 18(5):1201. 10.1158/1078-0432.CCR-11-0641 22186256 10.1158/1078-0432.CCR-11-0641 PMC3294063

[CR17] Dancey J (2010) mTOR signaling and drug development in cancer. Nat Rev Clin Oncol 7(4):209–219. 10.1038/NRCLINONC.2010.21 20234352 10.1038/nrclinonc.2010.21

[CR18] Duckett MM, Phung SK, Nguyen L, Khammanivong A, Dickerson E, Dusenbery K, Lawrence J (2020) The adrenergic receptor antagonists propranolol and carvedilol decrease bone sarcoma cell viability and sustained carvedilol reduces clonogenic survival and increases radiosensitivity in canine osteosarcoma cells. Veterinary Comparative Oncol 18(1):128–140. 10.1111/VCO.12560 10.1111/vco.12560 31778284

[CR19] Fasolo A, Sessa C (2012) Targeting mTOR pathways in human malignancies. Curr Pharm des 18(19):2766–2777. 10.2174/138161212800626210 22475451 10.2174/138161212800626210

[CR20] Guo YC, Chang CM, Hsu WL, Chiu SJ, Tsai YT, Chou YH, Hou MF, Wang JY, Lee MH, Tsai KL, Chang WC (2013) Indomethacin inhibits cancer cell migration via attenuation of cellular calcium mobilization. Molecules 8(6):6584–6596 10.3390/molecules18066584 PMC6269835 23736792

[CR21] Gustafson MP, Lin Y, Bleeker JS, Warad D, Tollefson MK, Crispen PL, Bulur PA, Harrington SM, Laborde RR, Gastineau DA, Leibovich BC, Cheville JC, Kwon ED, Dietz AB (2015) Intratumoral CD14+ cells and circulating CD14+HLA-DRlo/neg monocytes correlate with decreased survival in patients with clear cell renal cell carcinoma. Clin Cancer Res: off J Am Assoc Cancer Res 21(18):4224–4233. 10.1158/1078-0432.CCR-15-0260 10.1158/1078-0432.CCR-15-0260 25999436

[CR22] Haider R, Ricon-Becker I, Radin A, Gutman M, Cole SW, Zmora O, Ben-Eliyahu S, Haldar R, Ricon-Becker I, Radin A, Gutman M, Cole SW, Zmora O, Ben-Eliyahu S (2020) Perioperative COX2 and β-adrenergic blockade improves biomarkers of tumor metastasis, immunity, and inflammation in colorectal cancer: a randomized controlled trial. Cancer 126(17):3991–4001. 10.1002/cncr.32950 32533792 10.1002/cncr.32950

[CR23] Haldar R, Shaashua L, Lavon H, Lyons YA, Zmora O, Sharon E, Birnbaum Y, Allweis T, Sood AK, Barshack I, Cole S, Ben-Eliyahu S (2018) Perioperative inhibition of beta-adrenergic and COX2 signaling in a clinical trial in breast cancer patients improves tumor Ki-67 expression, serum cytokine levels, and PBMCs transcriptome. Brain, Behavior, Immun 73:294–309 10.1016/j.bbi.2018.05.014 29800703

[CR24] Heitz F, du Bois A, Harter P, Lubbe D, Kurzeder C, Vergote I, Plante M, Pfisterer J (2013) Impact of beta blocker medication in patients with platinum sensitive recurrent ovarian cancer-a combined analysis of 2 prospective multicenter trials by the AGO Study Group. NCIC-CTG EORTC-GCG. Gynecologic Oncol 129(3):463–466 10.1016/j.ygyno.2013.03.007 23500609

[CR25] Higgins JPT, Altman DG, Gøtzsche PC, Jüni P, Moher D, Oxman AD, Savović J, Schulz KF, Weeks L, Sterne JAC (2011) The cochrane collaboration’s tool for assessing risk of bias in randomised trials. BMJ. 10.1136/BMJ.D5928 22008217 10.1136/bmj.d5928 PMC3196245

[CR26] Hiller JG, Perry NJ, Poulogiannis G, Riedel B, Sloan EK (2018) Perioperative events influence cancer recurrence risk after surgery. Nat Rev Clin Oncol 15(4):205–218. 10.1038/NRCLINONC.2017.194 29283170 10.1038/nrclinonc.2017.194

[CR27] Hiller JG, Cole SW, Crone EM, Byrne DJ, Shackleford DM, Pang JB, Henderson MA, Nightingale SS, Ho KM, Myles PS et al (2020) Preoperative β-blockade with propranolol reduces biomarkers of metastasis in breast cancer: a phase II randomized trial. Clin Cancer Res 26(8):1803–1811 31754048 10.1158/1078-0432.CCR-19-2641

[CR28] Holmes WJM, Mishra A, Gorst C, Liew SH (2011) Propranolol as first-line treatment for rapidly proliferating infantile haemangiomas. J Plastic, Reconstructive Aesth Surg: JPRAS 64(4):445–451. 10.1016/J.BJPS.2010.07.009 10.1016/j.bjps.2010.07.009 20797926

[CR29] Howe LR, Brown AMC (2004) Wnt signaling and breast cancer. Cancer Biol Ther 3(1):36–41. 10.4161/CBT.3.1.561 14739782 10.4161/cbt.3.1.561

[CR30] Huettner FJ, Rooman I, Bouche G, Knebel P, Huesing J, Mihaljevic AL, Hackert T, Strobel O, Buechler MW, Diener MK (2020) Pancreatic resection with perioperative drug repurposing of propranolol and etodolac: trial protocol of the phase-II randomised placebo controlled PROSPER trial. BMJ Open. 10.1136/bmjopen-2020-040406 10.1136/bmjopen-2020-040406 PMC7528424 32998931

[CR31] Issa N, Byers S, Dakshanamurthy S (2013) Drug repurposing: translational pharmacology, chemistry, computers and the clinic. Curr Top Med Chem 13(18):2328–2336. 10.2174/15680266113136660163 24059462 10.2174/15680266113136660163 PMC11968090

[CR32] Kim HY, Jung YJ, Lee SH, Jung HJ, Pak K (2017) Is beta-blocker use beneficial in breast cancer? Meta-Anal Oncol 92(5):264–268. 10.1159/000455143 10.1159/000455143 28132057

[CR33] Knight JM, Rizzo JD, Logan BR, Wang T, Arevalo JMG, Ma J, Cole SW (2015) Low socioeconomic status, adverse gene expression profiles, and clinical outcomes in hematopoietic stem cell transplant recipients. Clin Cancer Res: off J Am Ass Cancer Res 22(1):69. 10.1158/1078-0432.CCR-15-1344 10.1158/1078-0432.CCR-15-1344 PMC4703514 26286914

[CR34] Knight JM, Rizzo JD, Hari P, Pasquini MC, Giles KE, D’Souza A, Logan BR, Hamadani M, Chhabra S, Dhakal B, Shah N, Sriram D, Horowitz MM, Cole SW (2020) Propranolol inhibits molecular risk markers in HCT recipients: a phase 2 randomized controlled biomarker trial. Blood Advnces 4(3):467–476 10.1182/bloodadvances.2019000765 PMC7013267 32027744

[CR35] Kokolus KM, Zhang Y, Sivik JM, Schmeck C, Zhu J, Repasky EA, Drabick JJ, Schell TD (2017) Beta blocker use correlates with better overall survival in metastatic melanoma patients and improves the efficacy of immunotherapies in mice. Oncoimmunology. 10.1080/2162402X.2017.1405205 29399407 10.1080/2162402X.2017.1405205 PMC5790362

[CR36] Léauté-Labrèze C, Hoeger P, Mazereeuw-Hautier J, Guibaud L, Baselga E, Posiunas G, Phillips RJ, Caceres H, Lopez Gutierrez JC, Ballona R, Friedlander SF, Powell J, Perek D, Metz B, Barbarot S, Maruani A, Szalai ZZ, Krol A, Boccara O, Voisard J-J (2015) A randomized, controlled trial of oral propranolol in infantile hemangioma. New England J Med 372(8):735–746 25693013 10.1056/NEJMoa1404710

[CR37] Lei Z, Yang W, Zuo Y (2021) Beta-blocker and survival in patients with lung cancer: a meta-analysis. PLoS ONE 16(2):e0245773. 10.1371/journal.pone.0245773 33592015 10.1371/journal.pone.0245773 PMC7886135

[CR38] Li J, Ji L, Chen J, Zhang W, Ye Z (2015) Wnt/β-Catenin signaling pathway in skin carcinogenesis and therapy. Biomed Res Int 2015:964842. 10.1155/2015/964842 26078973 10.1155/2015/964842 PMC4452418

[CR39] Li C, Li T, Tang R, Yuan S, Zhang W (2020) β-Blocker use is not associated with improved clinical outcomes in women with breast cancer: a meta-analysis. Biosci Rep, 40(6), BSR20200721. 10.1042/BSR20200721 10.1042/BSR20200721 PMC7303345 32436935

[CR40] Liao X, Chaudhary P, Qiu G, Che X, Fan L (2018) The role of propranolol as a radiosensitizer in gastric cancer treatment. Drug des Dev Ther 12:639–645. 10.2147/DDDT.S160865 10.2147/DDDT.S160865 PMC5880513 29636598

[CR41] Løfling LL, Støer NC, Sloan EK, Chang A, Gandini S, Ursin G, Botteri E (2022) β-blockers and breast cancer survival by molecular subtypes: a population-based cohort study and meta-analysis. Br J Cancer 127(6):1086–1096. 10.1038/s41416-022-01891-7 35725814 10.1038/s41416-022-01891-7 PMC9470740

[CR42] Logan CY, Nusse R (2004) The Wnt signaling pathway in development and disease. Annu Rev Cell Dev Biol 20:781–810. 10.1146/ANNUREV.CELLBIO.20.010403.113126 15473860 10.1146/annurev.cellbio.20.010403.113126

[CR43] Majidi A, Na R, Dixon-Suen S, Jordan SJ, Webb PM (2020) Common medications and survival in women with ovarian cancer: A systematic review and meta-analysis. Gynecol Oncol 157(3):678–685 32317171 10.1016/j.ygyno.2020.03.028

[CR44] Malik MA, Menon P, Rao KLN, Samujh R (2013) Effect of propranolol vs prednisolone vs propranolol with prednisolone in the management of infantile hemangioma: a randomized controlled study. J Pediatr Surg 48(12):2453–2459. 10.1016/J.JPEDSURG.2013.08.020 24314186 10.1016/j.jpedsurg.2013.08.020

[CR45] Melhem-Bertrandt A, Chavez-MacGregor M, Lei X, Brown EN, Lee RT, Meric-Bernstam F, Sood AK, Conzen SD, Hortobagyi GN, Gonzalez-Angulo AM (2011) Beta-blocker use is associated with improved relapse-free survival in patients with triple-negative breast cancer. J Clin Oncol 29(19):2645. 10.1200/JCO.2010.33.4441 21632501 10.1200/JCO.2010.33.4441 PMC3139371

[CR46] Modi ND, Tan JQE, Rowland A, Koczwara B, Kichenadasse G, McKinnon RA, Wiese MD, Sorich MJ, Hopkins AM (2020) The Influence of pre-existing beta-blockers use on survival outcomes in HER2 positive advanced breast cancer: pooled analysis of clinical trial data. Front Oncol 10:1130. 10.3389/fonc.2020.01130 32760671 10.3389/fonc.2020.01130 PMC7373122

[CR47] Montoya A, Amaya CN, Belmont A, Diab N, Trevino R, Villanueva G, Rains S, Sanchez LA, Badri N, Otoukesh S, Khammanivong A, Liss D, Baca ST, Aguilera RJ, Dickerson EB, Torabi A, Dwivedi AK, Abbas A, Chambers K, Nahleh Z (2017) Use of non-selective β-blockers is associated with decreased tumor proliferative indices in early stage breast cancer. Oncotarget 8(4):6446–6460 28031536 10.18632/oncotarget.14119 PMC5351644

[CR48] Montoya A, Varela-Ramirez A, Dickerson E, Pasquier E, Torabi A, Aguilera R, Nahleh Z, Bryan B (2019) The beta-adrenergic receptor antagonist propranolol alters mitogenic and apoptotic signaling in late stage breast cancer. Biomedical J 42(3):155–165. 10.1016/j.bj.2019.02.003 31466709 10.1016/j.bj.2019.02.003 PMC6717753

[CR49] Page MJ, McKenzie JE, Bossuyt PM, Boutron I, Hoffmann TC, Mulrow CD, Shamseer L, Tetzlaff JM, Akl EA, Brennan SE, Chou R, Glanville J, Grimshaw JM, Hróbjartsson A, Lalu MM, Li T, Loder EW, Mayo-Wilson E, McDonald S, Moher D (2021) The PRISMA 2020 statement: an updated guideline for reporting systematic reviews. BMJ. 10.1136/BMJ.N71 33782057 10.1136/bmj.n71 PMC8005924

[CR50] Pantziarka P, Bryan BA, Crispino S, Dickerson EB (2018) Propranolol and breast cancer—a work in progress. Ecancermedicalscience. 10.3332/ECANCER.2018.ED82 30034523 10.3332/ecancer.2018.ed82 PMC6027968

[CR51] Pasquier E, Ciccolini J, Carre M, Giacometti S, Fanciullino R, Pouchy C, Montero MP, Serdjebi C, Kavallaris M, André N (2011) Propranolol potentiates the anti-angiogenic effects and anti-tumor efficacy of chemotherapy agents: implication in breast cancer treatment. Oncotarget 2(10):797–809 22006582 10.18632/oncotarget.343 PMC3248157

[CR52] Peixoto R, Pereira M. de L, Oliveira M (2020) Beta-blockers and cancer: where are we? Pharmaceuticals, 13(6), 105 10.3390/ph13060105 PMC7345088 32466499

[CR53] Petrou T, Olsen HL, Thrasivoulou C, Masters JR, Ashmore JF, Ahmed A (2017) Intracellular calcium mobilization in response to ion channel regulators via a calcium-induced calcium release mechanism. J Pharmacol Exp Ther 360(2):378–387. 10.1124/JPET.116.236695/-/DC1 27980039 10.1124/jpet.116.236695 PMC5267512

[CR54] Powe DG, Voss MJ, Zänker KS, Habashy HO, Green AR, Ellis IO, Entschladen F (2010) Beta-blocker drug therapy reduces secondary cancer formation in breast cancer and improves cancer specific survival. Oncotarget 1(7):628 21317458 10.18632/oncotarget.197 PMC3248123

[CR55] Raimondi S, Botteri E, Munzone E, Cipolla C, Rotmensz N, DeCensi A, Gandini S (2016) Use of beta-blockers, angiotensin-converting enzyme inhibitors and angiotensin receptor blockers and breast cancer survival: Systematic review and meta-analysis. Int J Cancer 139(1):212–219 26916107 10.1002/ijc.30062

[CR56] Reyes-Corral M, Sørensen NM, Thrasivoulou C, Dasgupta P, Ashmore JF, Ahmed A (2019) Differential free intracellular calcium release by class II antiarrhythmics in cancer cell lsines. J Pharmacol Exp Ther 369(1):152–162. 10.1124/JPET.118.254375 30655298 10.1124/jpet.118.254375

[CR57] Ribatti D, Tamma R, Annese T (2020) Epithelial-mesenchymal transition in cancer: a historical overview. Transl Oncol 13(6):100773. 10.1016/J.TRANON.2020.100773 32334405 10.1016/j.tranon.2020.100773 PMC7182759

[CR58] Ricon-Becker I, Haldar R, Shabat Simon M, Gutman M, Cole SW, Ben-Eliyahu S, Zmora O (2023) Effect of perioperative COX-2 and beta-adrenergic inhibition on 5-year disease-free-survival in colorectal cancer: a pilot randomized controlled Colorectal Metastasis PreventIon Trial (COMPIT). Eur J Surg Oncol 49(3):655–661. 10.1016/j.ejso.2022.10.013

[CR59] Schatoff EM, Leach BI, Dow LE (2017) Wnt signaling and colorectal cancer. Current Colorectal Cancer Rep 13(2):101. 10.1007/S11888-017-0354-9 10.1007/s11888-017-0354-9 PMC5391049 28413363

[CR60] Scott OW, TinTin S, Cavadino A, Elwood JM (2024) Beta-blocker use and breast cancer outcomes: a meta-analysis. Breast Cancer Res Treat, 206(3), 443–463. 10.1007/s10549-024-07263-4 PT - Review 10.1007/s10549-024-07263-4 PMC11208256 38837086

[CR61] Shaashua L, Shabat-Simon M, Haldar R, Matzner P, Zmora O, Shabtai M, Sharon E, Allweis T, Barshack I, Hayman L, Arevalo J, Ma J, Horowitz M, Cole S, Ben-Eliyahu S (2017) Perioperative COX-2 and beta-adrenergic blockade improves metastatic biomarkers in breast cancer patients in a Phase-II randomized trial. Clin Cancer Res: off J Am Ass Cancer Res 23(16):4651–4661 10.1158/1078-0432.CCR-17-0152 PMC5559335 28490464

[CR62] Sondhi V, Patnaik SK (2013) Propranolol for infantile hemangioma (PINCH): an open-label trial to assess the efficacy of propranolol for treating infantile hemangiomas and for determining the decline in heart rate to predict response to propranolol. J Pediatr Hematol Oncol 35(7):493–499. 10.1097/MPH.0B013E3182A11658 23929318 10.1097/MPH.0b013e3182a11658

[CR63] Spera G, Fresco R, Fung H, Dyck JRB, Pituskin E, Paterson I, Mackey JR (2017) Beta blockers and improved progression-free survival in patients with advanced HER2 negative breast cancer: a retrospective analysis of the ROSE/TRIO-012 study. Ann Oncology: off J Eur Soc Med Oncol 28(8):1836–1841 10.1093/annonc/mdx264 28520849

[CR64] Spini A, Roberto G, Gini R, Bartolini C, Bazzani L, Donnini S, Crispino S, Ziche M (2019) Evidence of beta-blockers drug repurposing for the treatment of triple negative breast cancer: a systematic review. Neoplasma 66(6):963–970. 10.4149/neo_2019_190110N34 31607128 10.4149/neo_2019_190110N34

[CR65] Tanaka H, Kuroda A, Marusawa, et al (1987) Structure of FK 506: a novel immunosuppressant isolated from Streptomyces. J Am Chem Soc. 10.1021/ja00250a050 2445067

[CR66] Tewarie IA, Senders JT, Hulsbergen AFC, Kremer S, Broekman MLD (2021) Beta-blockers and glioma: a systematic review of preclinical studies and clinical results. Neurosurg Rev 44(2):669–677. 10.1007/s10143-020-01277-4 32172480 10.1007/s10143-020-01277-4 PMC8035104

[CR67] Trompet S, De Craen AJM, Mooijaart S, Stott DJ, Ford I, Sattar N, Jukema W, Westendorp RGJ (2009) High innate production capacity of proinflammatory cytokines increases risk for death from cancer: Results of the PROSPER study. Clin Cancer Res 15(24):7744–7748 19996221 10.1158/1078-0432.CCR-09-2152

[CR68] Uleri A, Baboudjian M, Tedde A, Rajwa P, Pradere B, Gallioli A, Breda A, Ploussard G (2024) The association between beta-blockers use and prostate cancer mortality: a mini systematic review and meta-analysis. Prostate 84(1):3–7. 10.1002/pros.24623 37710384 10.1002/pros.24623

[CR69] Vacher S, Castagnet P, Chemlali W, Lallemand F, Meseure D, Pocard M, Bieche I, Perrot-Applanat M (2018) High AHR expression in breast tumors correlates with expression of genes from several signaling pathways namely inflammation and endogenous tryptophan metabolism. PLoS ONE 13(1):e0190619. 10.1371/JOURNAL.PONE.0190619 29320557 10.1371/journal.pone.0190619 PMC5761880

[CR70] Vercellino N, Romanini MV, Pelegrini M, Rimini A, Occella C, Dalmonte P (2013) The use of propranolol for complicated infantile hemangiomas. Int J Dermatol 52(9):1140–1146. 10.1111/J.1365-4632.2012.05795.X 23829783 10.1111/j.1365-4632.2012.05795.x

[CR71] Wang Q, Symes AJ, Kane CA, Freeman A, Nariculam J, Munson P, Thrasivoulou C, Masters JRW, Ahmed A (2010) A novel role for Wnt/Ca2+ signaling in actin cytoskeleton remodeling and cell motility in prostate cancer. PLoS ONE 5(5):e10456. 10.1371/JOURNAL.PONE.0010456 20454608 10.1371/journal.pone.0010456 PMC2864254

[CR72] Wang T, Li Y, Lu HL, Meng QW, Cai L, Chen XS (2015) β-Adrenergic receptors: new target in breast cancer. Asian Pacific J Cancer Prevention: APJCP 16(18):8031–8039. 10.7314/apjcp.2015.16.18.8031 10.7314/apjcp.2015.16.18.8031 26745035

[CR73] Wang J, Lu S, Meng Y, Fu W, Zhou X (2022) Beta adrenergic blockade and clinical outcomes in patients with colorectal cancer: a systematic review and meta-analysis. Eur J Pharmacol 929:175135. 10.1016/j.ejphar.2022.175135 35798050 10.1016/j.ejphar.2022.175135

[CR74] Weberpals J, Jansen L, Carr PR, Hoffmeister M, Brenner H (2016) Beta blockers and cancer prognosis - The role of immortal time bias: a systematic review and meta-analysis. Cancer Treat Rev 47:1–11. 10.1016/J.CTRV.2016.04.004 27179912 10.1016/j.ctrv.2016.04.004

[CR75] Weiss S, Oz S, Benmocha A, Dascal N (2013) Regulation of cardiac L-type Ca2+ channel CaV1.2 via the β-adrenergic-cAMP-protein kinase A pathway: old dogmas, advances, and new uncertainties. Circulation Res. 10.1161/CIRCRESAHA.113.301781 23948586 10.1161/CIRCRESAHA.113.301781

[CR76] Wen ZY, Gao S, Gong TT, Jiang YT, Zhang JY, Zhao YH, Wu QJ (2021) Post-diagnostic beta blocker use and prognosis of ovarian cancer: a systematic review and meta-analysis of 11 cohort studies With 20,274 patients. Front Oncol 11:665617. 10.3389/fonc.2021.665617 34221981 10.3389/fonc.2021.665617 PMC8247638

[CR77] Wolter JK, Wolter NE, Blanch A, Partridge T, Cheng L, Morgenstern DA, Podkowa M, Kaplan DR, Irwin MS (2014) Anti-tumor activity of the beta-adrenergic receptor antagonist propranolol in neuroblastoma. Oncotarget 5(1):161–172 24389287 10.18632/oncotarget.1083 PMC3960198

[CR78] Xia Y, Sun M, Huang H, Jin WL (2024) Drug repurposing for cancer therapy. Signal Transduction Targeted Ther. 10.1038/s41392-024-01808-1 10.1038/s41392-024-01808-1 PMC11026526 38637540

[CR79] Yan X, Liu P, Li D, Hu R, Tao M, Zhu S, Wu W, Yang M, Qu X (2022) Novel evidence for the prognostic impact of beta-blockers in solid cancer patients receiving immune checkpoint inhibitors. Int Immunopharmacology. 10.1016/j.intimp.2022.109383 10.1016/j.intimp.2022.109383 36330916

[CR80] Yang J, Zhang S, Jiang W (2023) Impact of beta blockers on breast cancer incidence and prognosis. Clin Breast Cancer 23(6):664-671.e21. 10.1016/j.clbc.2023.05.014 37353431 10.1016/j.clbc.2023.05.014

[CR81] Yap A, Lopez-Olivo MA, Dubowitz J, Pratt G, Hiller J, Gottumukkala V, Sloan E, Riedel B, Schier R (2018) Effect of beta-blockers on cancer recurrence and survival: a meta-analysis of epidemiological and perioperative studies. Br J Anaesth 121:45–57. 10.1016/j.bja.2018.03.024 29935594 10.1016/j.bja.2018.03.024

[CR82] Zhong S, Yu D, Zhang X, Chen X, Yang S, Tang J, Zhao J, Wang S (2016) β-Blocker use and mortality in cancer patients: systematic review and meta-analysis of observational studies. Eur J Cancer Prevention: off J Eur Cancer Prevention Org (ECP) 25(5):440–448. 10.1097/CEJ.0000000000000192 10.1097/CEJ.0000000000000192 26340056

[CR83] Zhou L, Li Y, Li X, Chen G, Liang H, Wu Y, Tong J, Ouyang W (2016) Propranolol attenuates surgical stress-induced elevation of the regulatory T cell response in patients undergoing radical mastectomy. J Immunol 196(8):3460–3469. 10.4049/jimmunol.1501677 26969754 10.4049/jimmunol.1501677

