# Supplementary material for: Does propranolol have a role in cancer treatment? A systematic review of the epidemiological and clinical trial literature on beta-blockers
Source: J Cancer Res Clin Oncol. 2025 Jul 12;151(7):212. doi: 10.1007/s00432-025-06262-2 (PMC12255574; doi:10.1007/s00432-025-06262-2)
Supplement: Supplementary file 1 — Supplementary Material 1 [file 432_2025_6262_MOESM1_ESM.docx]

Table A: Full search strategy.

| **#** | **MEDLINE** | **Embase.com** | **Global Health** | **Web of Science** | **Cochrane Library** |
| --- | --- | --- | --- | --- | --- |
| 1 | (beta-block* or propranolol).tw. | (beta-block* or propranolol).tw. | (beta-block* or propranolol).tw | **(TI=(beta-block* OR propranolol) OR AB=(beta-block* OR propranolol))** | (beta-block* or propranolol):ti,ab,kw |
| 2 | (cancer* or tumor* or tumour* or oncolog* or malignan*).tw. | (cancer* or tumor* or tumour* or oncolog* or malignan*).tw. | (cancer* or tumor* or tumour* or oncolog* or malignan*).tw. | TI=(cancer* or tumor* or tumour* or oncolog* or malignan*) OR AB=(cancer* or tumor* or tumour* or oncolog* or malignan*) | (cancer* or tumor* or tumour* or oncolog* or malignan*):ti,ab,kw |
| 3 | (Clinical trial or meta-analysis or randomi*ed controlled trial or review or systematic review).pt. | (Clinical trial or meta-analysis or randomi*ed controlled trial or review or systematic review).pt. | #1 AND #2 | DT==("Clinical trial" or "meta-analysis" or "randomi*ed controlled trial" or "review" or "systematic review") | #1 AND #2  Limits applied: Cochrane Reviews and Trials |
| 4 | #1 AND #2 AND #3 | #1 AND #2 AND #3 | Limit 3 to (english language and "systematic review" and journal article) | #1 AND #2 AND #3 |  |
| 5 | Limit 4 to (english language and humans) | Limit 4 to (english language and humans) |  | (#1 AND #2 AND #3) AND LA==(“English”) |  |
| 6 | #1 AND #2 AND #3 AND #4 AND #5 | #1 AND #2 AND #3 AND #4 AND #5 |  | #1 AND #2 AND #3 AND #4 AND #5  N=750 |  |

*Note:* .tw = text word (denotes searching for a key word in the title and abstract), .pt = publication type, TI = title, AB = abstract, DT = document type, LA = language, exp *= the subject heading has been focussed (limiting to articles where the selected subject heading is a major concept of the article), * = truncation.
